# Cause-Specific Excess Mortality During the COVID-19 Pandemic (2020–2021) in 12 Countries of the C-MOR Consortium

**DOI:** 10.1007/s44197-024-00242-4

**Published:** 2024-05-22

**Authors:** Victoria Virginia Beeks, Souzana Achilleos, Annalisa Quattrocchi, Chryso Th. Pallari, Elena Critselis, Pascale Salameh, Mohammad Reza Rahmanian Haghighi, Jose Manuel Rodriguez-Llanes, Giuseppe Ambrosio, Andreas Artemiou, John Gabel, Catherine Marie Bennett, Joseph Cuthbertson, Claudia Zimmermann, Eva Susanna Schernhammer, Antonio José Leal Costa, Luciana Freire de Carvalho, Jackeline Christiane Pinto Lobato, Maria Athanasiadou, Julia Alison Critchley, Lucy Pollyanna Goldsmith, Levan Kandelaki, Natalya Glushkova, Kairat Davletov, Yuliya Semenova, Ivan Erzen, Olesia Verstiuk, Dimos Alekkou, Antonis Polemitis, Andreas Charalambous, Christiana A. Demetriou, The C-MOR Consortium

**Affiliations:** 1 Department of Primary Care and Population Health, University of Nicosia Medical School, Nicosia, Cyprus; 2 PlanAdapt, Berlin, Germany; 3 Department of Cardiology, University of Perugia School of Medicine, Perugia, Italy; 4 Department of Information Technologies, University of Limassol, Limassol, Cyprus; 5 Massachusetts Institute of Technology, Cambridge, MA USA; 6 School of Health and Social Development, Institute for Health Transformation, Deakin University, Waurn Ponds, Australia; 7 Monash University Disaster Resilience Initiative, Monash University, Melbourne, Australia; 8 Department of Epidemiology, Center for Public Health, Medical University of Vienna, Vienna, Austria; 9 Instituto de Estudos em Saúde Coletiva, Universidade Federal do Rio de Janeiro, Rio de Janeiro, Brazil; 10 Departamento de Epidemiologia do Instituto de Saúde Coletiva, Universidade Federal Fluminense, Niterói, Brazil; 11 Health Monitoring Unit, Cyprus Ministry of Health, 1 Prodromou & 17 Chilonos Street, 1448 Nicosia, Cyprus; 12 Population Health Research Institute, St George’s University of London, London, UK; 13 Medical Statistics, National Center for Disease Control and Public Health, Tbilisi, Georgia; 14 Health Research Institute, Al-Farabi Kazakh National University, Almaty, Kazakhstan; 15 Asfendiyarov Kazakh National Medical University, Almaty, Kazakhstan; 16 School of Medicine, Nazarbayev University, Astana, Kazakhstan; 17 School of Public Health, National Institute of Public Health, Ljubljana, Slovenia; 18 Department of Medical Science, University of Nicosia, Nicosia, Cyprus; 19 Department of Psychology, University of Nicosia, Nicosia, Cyprus; 20 University of Nicosia, Nicosia, Cyprus; 21 University of Nicosia Medical School, Nicosia, Cyprus

**Keywords:** Cause-specific mortality, COVID-19, Cardiovascular diseases, Cancer, Respiratory tract infections, Public health measures

## Abstract

**Background:**

This study investigated cause-specific mortality rates in 12 countries during the COVID-19 pandemic in 2020 and 2021.

**Methods:**

We collected weekly cause-specific mortality data from respiratory disease, pneumonia, cardiovascular disease (CVD) and cancer from national vital statistic databases. We calculated excess mortality for respiratory disease (excluding COVID-19 codes), pneumonia, and CVD in 2020 and 2021 by comparing observed weekly against expected mortality based on historical data (2015–2019), accounting for seasonal trends. We used multilevel regression models to investigate the association between country-level pandemic-related variables and cause-specific mortality.

**Results:**

Significant reductions in cumulative mortality from respiratory disease and pneumonia were observed in 2020 and/or 2021, except for Georgia, Northern Ireland, Kazakhstan, and Ukraine, which exhibited excess mortality for one or both causes. Australia, Austria, Cyprus, Georgia, and Northern Ireland experienced excess cumulative CVD mortality in 2020 and/or 2021. Australia, Austria, Brazil, Cyprus, Georgia, Northern Ireland, Scotland and Slovenia, experienced increased crude cumulative cancer mortality during 2020 and/or 2021 compared to previous years. Among pandemic-related variables, reported COVID-19 incidence was negatively associated with increased cancer mortality, excess respiratory, (2020) and pneumonia (2021) mortality, and positively associated with respiratory and CVD mortality (2021). Stringency of control measures were negatively associated with excess respiratory disease, CVD, and increased cancer mortality (2021).

**Conclusions:**

This study provides evidence of substantial excess mortality from CVD, and notable reductions in respiratory disease and pneumonia in both years across most countries investigated. Our study also highlights the beneficial impact of stringent control measures in mitigating excess mortality from most causes in 2021.

**Supplementary Information:**

The online version contains supplementary material available at 10.1007/s44197-024-00242-4.

## Introduction

The severe acute respiratory syndrome coronavirus 2 triggered a pandemic and led to a global public health emergency and response of historical magnitude [1]. The disease, named coronavirus-19 (COVID-19), infected millions, and has had extensive impacts on disease management and mortality, directly through community spread and indirectly through disruptions in screening, diagnosis, and treatment processes [2]. Healthcare systems were compelled to restructure and adapt to the novel disease, while ensuring continuous provision of routine and emergency care for other conditions. The interruption of vital healthcare services, including referrals, diagnoses, and treatments, has been suggested to contribute to excess cardiovascular (CVD) [3] and cancer mortality [4]. Several studies have reported excess mortality across CVD, cancer, and respiratory disease causes during the pandemic [5–7].

To date, research on the impact of the COVID-19 pandemic on mortality has primarily focused on all-cause mortality rates [8]. Research on cause-specific mortality remains limited and fragmented [5, 7], often analysing data from single countries [6]. In an effort to achieve a better understanding of the pandemic and improve future public health response, the COVID-19 MORtality (C-MOR) Consortium was established, aiming to investigate the impact of the pandemic on mortality. The consortium strives to foster a global reach, transcending geographical limitations, by utilizing data from national primary sources across countries worldwide [9, 10]. The present analysis focuses on examining the magnitude of excess cause-specific mortality during the COVID-19 pandemic in 12 countries of the C-MOR consortium. This study also aims to identify pandemic-related predictors of excess cause-specific mortality. The analysis aims to provide a deeper understanding of the burden of the pandemic on various causes of death, generating valuable knowledge for informing decision-making processes in future pandemics.

## Methods

### Data Collection

#### Mortality Data Collection

We collected mortality data from 12 countries participating in the C-MOR consortium (Australia, Austria, Brazil, Cyprus, England and Wales, Georgia, Kazakhstan, Northern Ireland, Scotland, Slovenia, Ukraine, and the United States of America (USA)), between June–September 2022, from national vital statistic databases, either publicly available or with restricted access (Supplementary Table S1). Data collection several months after the study period accounted for reporting delays. We collected weekly mortality data between 2015 and 2021 for all ages and both genders from each country, for the following causes: respiratory disease (ICD-10: J00-J99), pneumonia (ICD-10: J12.0-J18.9), CVD (ICD-10: l00-l09, l11, l20 -l51, l10, l12, l60-l69, l70-l78 & l80-l99), and cancer (ICD-10: C00-C97). Notably, COVID-19 codes (ICD-10: U07.1 and U07.2) were excluded from respiratory disease mortality, as countries in the analysis separated them distinctly. Variability in how these codes were used to register COVID-19 deaths precluded analysis of COVID-19 specific mortality in this study. Weeks were defined as ISO week (Monday-Sunday), Epi week (Sunday-Saturday), or national counting week, varying by country. Data for all causes were available across all participating countries except for England and Wales (only respiratory), Kazakhstan (respiratory, pneumonia), Scotland (respiratory, cancer), and the USA (respiratory, CVD).

#### Pandemic-Related Variable Collection

To identify variables potentially associated with excess mortality in the participating countries, we collected data on pandemic-related variables (reported weekly), including incidence of reported COVID-19 cases, stringency of control measures and vaccination rates as percentage of population fully vaccinated, from publicly available and reliable sources, outlined in Supplementary Table S2.

### Statistical Analysis

#### Observed Mortality Rates

We calculated mortality rates using total mid-year population estimates obtained by the World Bank for all participating countries [11], except for the United Kingdom (UK) (population sourced from the Office for National Statistics [12]), and Cyprus (Republic of Cyprus population obtained from Eurostat [13]).

Weekly crude mortality rates (CMRs) were calculated for the total population using Eq. (1)

1 $$Crude \; mortality \; rate \; \left( {CMR} \right)_{y,w} = \frac{{D_{y,w} }}{{P_{y} /N_{w} }} \times 100,000$$

where, *D*_*y,w*_ represents the number of deaths in year* y* and week *w, P*_*y*_ represents the mid-year population in year* y*, while *N*_*w*_ represents the number of weeks in the year.

As a preliminary investigation, we analyzed the relationship between observed weekly average CMRs from 2015 to 2019 and 2020 to 2021 using a generalized estimating equation (GEE) model with a Poisson distribution, for each cause. CMRs were rounded to the nearest integer. We used the Wald test to assess the significance of the estimated model parameters, with a significance level at *p* < *0.05.*

#### Excess Mortality

We estimated expected 2020 and 2021 mortality rates based on 2015–2019 historical data using a Generalized Linear Model (GLM) with a Poisson Regression, accounting for seasonal trends, as explained elsewhere [10]. We applied the model to the total population of each country, for respiratory, pneumonia, and CVD causes, separately. We estimated expected mortality rates for complete weeks, excluding truncated weeks (supplementary Table S3). We determined statistical significance using 95% confidence intervals estimated by the model. We present weekly results of observed vs expected CMRs graphically using z-scores obtained using Eq. (2). Z-scores between -2 and + 2 are considered normal, while z-scores > 4 or < -4 signify a substantial increase or decrease, respectively.

2 $$Z{\text{-}}score = \frac{Excess \; CMRs}{{SD}}$$

where, *SD* = standard deviation of residuals.

Subsequently, we calculated cumulative excess mortality for 2020 and 2021 by subtracting cumulative expected mortality from the cumulative observed rates, for each cause. We determined statistical significance using the 95% CIs estimated by the model.

The data of weekly cancer mortality was a poor fit in the GLM with R^2^ values spanning from 0.02 to 0.38  across countries, suggesting potential unreliability in the findings. Consequently, we excluded cancer entirely from the time-series analysis.

For cancer, we calculated cumulative excess mortality (as % change) by subtracting the total average 2015–2019 CMRs from the total 2020 and 2021 CMRs. Additionally, we used a joinpoint regression to calculate the percent change in CMRs across trimesters, allowing the identification of significant changes in cancer mortality trends [14]. We set a maximum of 5 joinpoints as a parameter for the regression, and for each country, we retained the model with the best fit (most pertinent number of joinpoints). We also considered the use of weekly CMRs for the latter analysis; however the large volume of data points precluded the execution of a joinpoint regression within the software.

#### Associations of Pandemic-Related Variables with Excess Mortality

We utilized multilevel models to examine pandemic-related variables, including weekly COVID-19 incidence, stringency of control measures, and the percentage of fully vaccinated individuals, with country as a random effect, the variables as fixed effects, and the method of restricted maximum likelihood. We ran the model separately for 2020 (excluding vaccinations) and for 2021 for each cause. We considered interactions among pandemic-related variables but retained them only if they improved the model fit*.* In all models, we used excess mortality z-scores as the outcome, excluding z-scores > 15 as outliers identified using bag plots. For cancer, we used the percent change in observed 2020 and 2021 mortality rate versus 2015–2019 as the outcome.

We set nominal significance at 0.05, acknowledging that this is an exploratory study. Therefore, all p-values should be interpreted accordingly.

We conducted statistical analyses using R Statistical Software, version 4.2.1 (The R Foundation for Statistical Computing, Vienna, Austria), except for  joinpoint regression, for which we used Joinpoint Trend Analysis Software, version 5.0.2. We produced graphical representations in R statistical Software, version 4.2.1., STATA/BE 17.0, and Joinpoint Trend Analysis Software, version 5.0.2.

## Results

### Weekly Observed Mortality Rates for Total Population

Our results reveal that average weekly mortality rates varied by cause, country and time. Table 1 displays mean values, coefficients and p-values of observed weekly average mortality rates for each cause in 2020 and 2021, compared to 2015–2019. Supplementary Figures S1–S4 offer graphical comparisons.

**Table 1:** Observed weekly average mortality rates for respiratory disease (in 12 countries), pneumonia, cardiovascular disease and cancer (in 9 countries) in 2020 and 2021 compared to 2015–2019 (per 100 000 population)

Country	Respiratory disease					Pneumonia				
	2015–2019	2020		2021		2015–2019	2020		2021	
	Mean	Mean	Coef.	Mean	Coef.	Mean	Mean	Coef.	Mean	Coef.
Australia	58.4	47.9	− 0.2***	50.6	− 0.14 ***	11.1	8.3	− 0.29***	8.2	− 0.3***
Austria	56.7	55.1	− 0.03***	44.6	− 0.24***	12.7	10.3	− 0.21**	7.8	− 0.48*
Brazil	74.6	70.7	− 0.05*	66.8	− 0.11***	38.4	31.9	− 0.18***	31.6	− 0.20***
Cyprus	66.2	70.5	0.6	57.5	− 0.14*	7.7	6.8	− 0.12	7.9	0.04
England & Wales	126.3	105.7	− 0.17**	90.4	− 0.33***					
Georgia	73.5	108.2	0.39***	97.6	0.28***	32.9	73.4	0.78***	64.7	0.67***
Kazakhstan	77.9	108.0	0.32	105.7	0.3***	16.9	46.7	1.1***	30.0	0.56***
Northern Ireland	241.5	284.9	0.17**	284.9	0.17*	39.6	22.2	− 0.58***	21.1	− 0.63***
Scotland	131.3	101.8	− 0.25***	98.6	− 0.27***					
Slovenia	57.5	38.3	− 0.39***	31.4	− 0.61***	25.3	12.9	− 0.64***	11.5	− 0.79***
Ukraine	17.4	23.5	0.31***	64.6	1.31***	12.6	20.6	0.47***	38.5	1.12***
USA	167.0	164.9	− 0.01	148.6	− 0.12***					

Country	Cardiovascular disease					Cancer				
	2015–2019	2020		2021		2015–2019	2020		2021	
	Mean	Mean	Coef.	Mean	Coef.	Mean	Mean	Coef.	Mean	Coef.
Australia	61.6	53.7	− 0.14***	53.6	− 0.14***	187.6	189.8	0.01*	189.9	0.01*
Austria	379.8	371.8	− 0.2	348.9	− 0.8***	232.0	238.5	0.03**	230.1	− 0.08
Brazil	169.3	168.1	− 0.01	172.0	0.02	104.2	107.0	0.03***	105.4	0.01**
Cyprus	220.8	210.8	− 0.05	203.5	− 0.08	161.5	172.3	0.06*	175.0	0.07*
Georgia	533.4	593.0	0.99**	583.7	0.10***	181.8	212.2	0.15***	195.0	0.07***
Northern Ireland	195.5	193.7	− 0.01	190.2	− 0.03	239.4	240.9	0.01	242.2	0.01
Scotland						299.0	301.7	0.01	303.0	0.01
Slovenia	377.5	371.4	− 0.02	353.3	− 0.07	300.9	307.3	0.02	292.2	− 0.03
Ukraine	1228.0	1797.2	0.40***	193.4	− 1.85***	178.2	174.6	− 0.01	174.1	− 0.02
USA	239.7	259.4	0.08***	254.4	0.06***					

p < 0.001***, p < 0.01**, p < 0.05*

### Weekly Observed vs. Expected Comparisons for Total Population

**Fig. 1:**
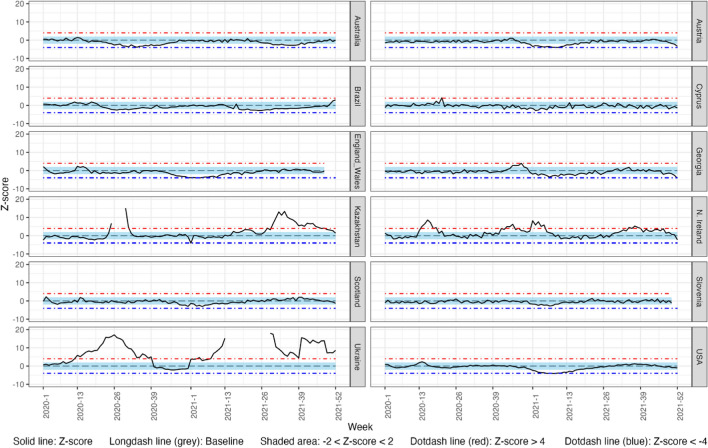
Weekly z-score of mortality rates from respiratory disease for the total population in 2020 and 2021 in 12 countries of the C-MOR consortium. Complete figures of Kazakhstan and Ukraine are presented in supplementary figures S5 and S6

**Fig. 2:**
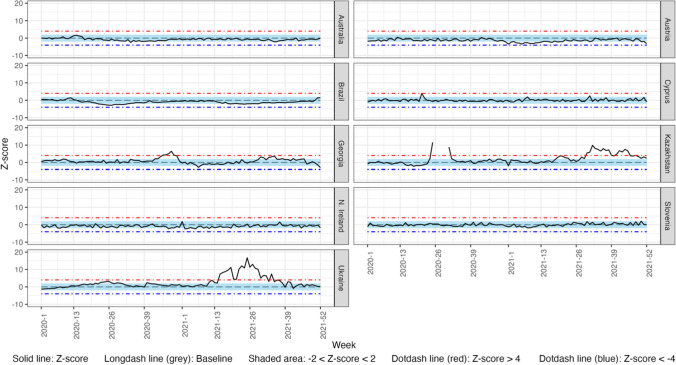
Weekly z-score of mortality rates from pneumonia for the total population in 2020 and 2021 in 9 countries of the C-MOR consortium. A complete figure of Kazakhstan is presented in supplementary figure S7

**Fig. 3:**
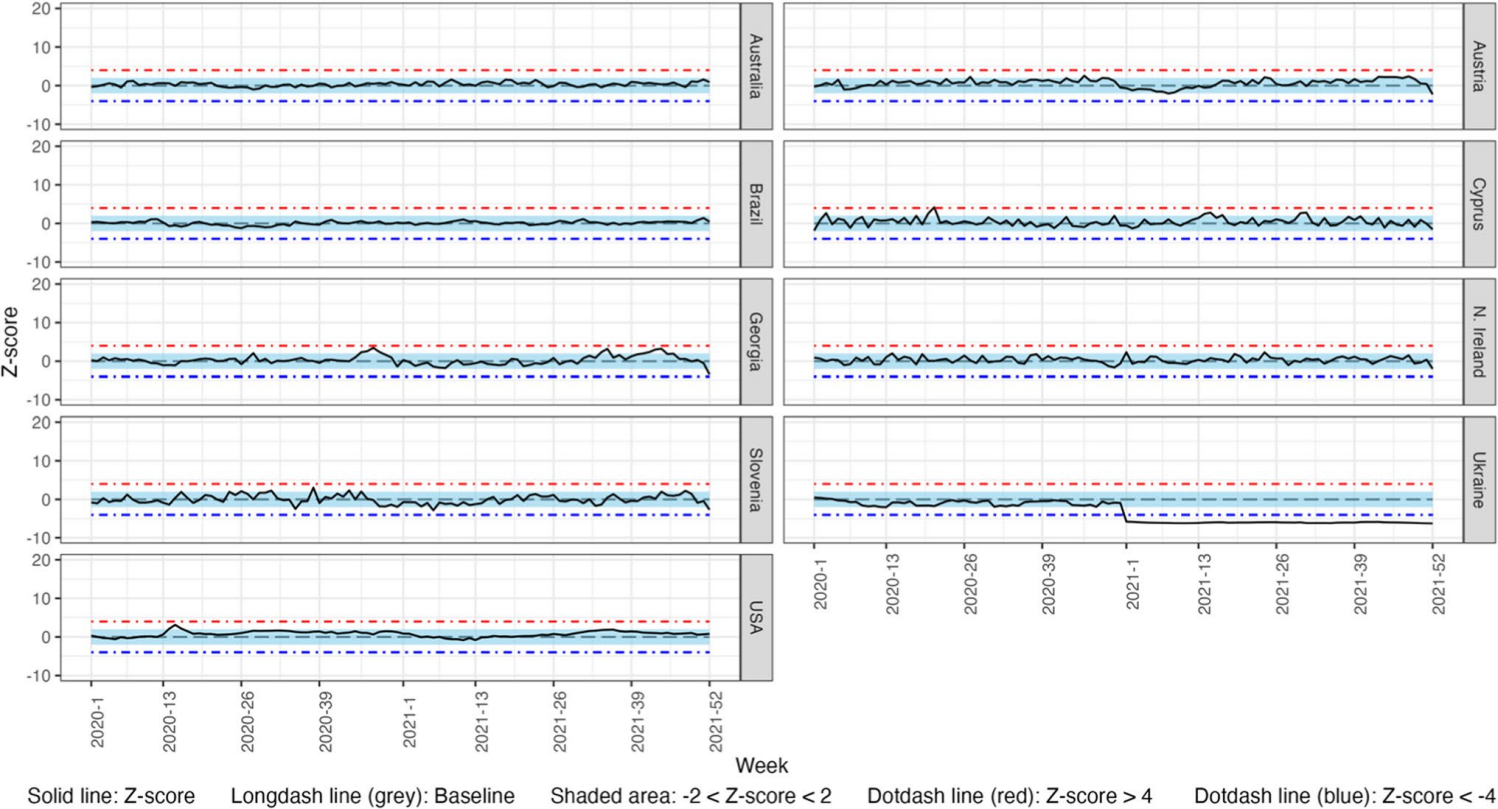
Weekly z-score of mortality rates from cardiovascular disease for the total population in 2020 and 2021 in 9 countries of the C-MOR consortium

Figures 1, 2 and 3 depict weekly CMR z-scores from Week 1 2020 to Week 52 2021 for the total population, for respiratory disease, pneumonia, and CVD. Supplementary Table S4 details weeks of substantial cause-specific mortality change in countries.

### Cumulative Excess Mortality for the Total Population

Cumulative observed and expected cause-specific mortality rates for 2020 and 2021 (excluding cancer) are presented in supplementary Tables S5–S7. Supplementary Figures S8–S10 display cumulative excess mortality (except cancer) for the same years.

#### Respiratory Disease

In 2020 and 2021, Northern Ireland, Kazakhstan and Ukraine experienced significant excess cumulative respiratory mortality, while Australia, Austria, Brazil, England and Wales, Scotland and Slovenia demonstrated significant decreases. In 2021, Cyprus, Georgia and the USA experienced significant decreases in respiratory mortality, while no significant changes were observed in 2020.

#### Pneumonia

Kazakhstan and Ukraine presented significant excess pneumonia mortality, while Australia, Austria, Brazil, and Northern Ireland presented significant decreases in both years. Georgia experienced excess pneumonia mortality, whereas Cyprus and Slovenia experienced significant decreases only in 2020, with no significant changes in 2021.

#### Cardiovascular Disease

In 2020, Austria, Cyprus, Georgia, and Northern Ireland experienced excess CVD mortality. In 2021, Australia, Austria, Cyprus, and Northern Ireland also experienced excess CVD mortality. Ukraine experienced a significant decrease in cumulative CVD mortality, in both years, while no significant changes were presented in Brazil and Slovenia.

#### Cancer

Supplementary Table S8 displays cumulative crude cancer mortality in 2020 and 2021 compared to 2015–2019. Australia, Brazil, Cyprus, Georgia, Northern Ireland, and Scotland, demonstrated increased cancer mortality in both years, compared to 2015–2019. Austria and Slovenia demonstrated increased cancer mortality in 2020 and decreased cancer mortality in 2021, while Ukraine experienced decreases in both years compared to baseline. Supplementary Figure S11 presents the joinpoint regression results. Brazil experienced significant increases (+ 0.51%) in trimesters 1–19 (01/2015–09/2019) while significant decreases (− 0.44%) were demonstrated in trimesters 19–28 (09/2019–12/2021). Georgia presented significant increases (+ 1.31%) throughout trimesters 1–23 (01/2015–09/2020), whereas significant decreases were noticed in trimesters 23–28 (09/2020–12/2021) (− 2.39%). Cyprus demonstrated significant increases (+ 0.5%) throughout all trimesters, while the other countries did not exhibit significant changes.

### Pandemic-Related Associations of Excess Mortality

Summary statistics of pandemic-related variables by country are detailed in supplementary Table S9. Results from the pandemic-related associations of excess mortality are presented in Table 2.

**Table 2:** Multilevel model results on the ability of pandemic related variables to predict excess mortality rates for respiratory disease (in 12 countries), pneumonia, cardiovascular disease and cancer (in 9 countries) during 2020 and 2021

Predictors	Respiratory disease				Pneumonia			
	2020		2021		2020		2021	
	Estimates	CI	Estimates	CI	Estimates	CI	Estimates	CI
Weekly incidence of COVID-19 (3-week lag)	− 0.74***	− 1.00 to − 0.49	0.28**	0.05 to 0.50	− 0.11	− 0.41 to 0.20	− 0.69**	− 1.20 to − 0.17
Stringency of control measures (3-week lag)	0.04	− 0.12 to 0.20	− 0.56***	− 0.77 to − 0.36	− 0.15	− 0.33 to 0.03	− 0.03	− 0.30 to 0.24
Number of fully vaccinated per 100 population (2-week lag)			0.41***	0.19 to 0.63			0.19	− 0.11 to 0.49
**Random effects**	**2020**		**2021**		**2020**		**2021**	
ICC	0.45		0.75		0.35		0.60	
Observations	511		585		388		452	
Marginal R^2^ /conditional R^2^	0.087/0.502		0.070/0.764		0.063/0.389		0.078/0.630	

Predictors	CVD				Cancer			
	2020		2021		2020		2021	
	Estimates		CI		Estimates		CI	
Weekly incidence of COVID-19 (3-week lag)	0.19	− 0.01 to 0.39	0.13**	0.00 to 0.25	− 2.83**	− 4.61 to − 1.06	− 1.65	− 3.96 to 0.67
Stringency of control measures (3-week lag)	− 0.10	− 0.24 to 0.04	− 0.29***	− 0.39 to − 0.19	1.49**	0.44 to 2.54	− 1.83**	− 2.97 to − 0.69
Number of fully vaccinated per 100 population (2-week lag)			0.10	− 0.01 to 0.21			1.40**	0.16 to 2.64
**Random effects**	**2020**		**2021**		**2020**		**2021**	
ICC	0.39		0.87		0.23		0.13	
Observations	393		459		320		410	
Marginal R^2^ /conditional R^2^	0.021/0.404		0.026/0.877		0.072/0.282		0.089/0.206	

Excess mortality rate z-scores were used for all causes, except for cancer where the percent change of 2020 or 2021 observed compared to 2015–2019 observed mortality rate was used. Participating countries: Australia, Austria, Brazil, Cyprus, Georgia, England and Wales (for respiratory disease only), Kazakhstan (for respiratory and pneumonia), Northern Ireland, Scotland (for respiratory disease and cancer), Slovenia, Ukraine. Interaction terms were included in the models only when they improved model fit. A significant association was found between the interaction of stringency and weekly incidence of COVID-19 with pneumonia mortality (2020; − 0.42**, 2021; 1.02***), and with CVD mortality (2020; − 0.38**)

*CI* confidence intervals, *ICC* intra-class correlation, *CVD* cardiovascular disease

p < 0.001***, p < 0.01**, p < 0.05*

#### Respiratory Disease

In the 2020 model, weekly incidence of reported COVID-19 cases was negatively associated with excess respiratory disease mortality z-scores, while stringency of control measures showed no independent association. In the 2021 model, weekly incidence of reported COVID-19 cases was positively associated with excess respiratory mortality, while stringency of control measures had a negative association. The number of people fully vaccinated was independently, positively associated with excess respiratory mortality.

#### Pneumonia

In the 2020 model, no significant associations were reported for weekly incidence of COVID-19 and stringency of control measures with excess pneumonia mortality. However, a negative association was observed for the interaction between these variables. In the 2021 model, weekly incidence of COVID-19 cases was independently, negatively associated with excess pneumonia mortality. There were no significant associations between stringency of control measures, fully vaccinated individuals, and excess pneumonia mortality; however, a significant interaction was observed between weekly COVID-19 incidence and control measure stringency.

#### Cardiovascular Disease

In 2020, we did not observe any significant independent associations were observed for CVD mortality and the included variables, however, we found a negative association between COVID-19 incidence and CVD mortality through interaction with stringency of control measures. In 2021, we observed a positive association between weekly COVID-19 incidence and CVD mortality, whereas stringency of control measures negatively affected CVD mortality. No associations were found between fully vaccinated individuals and CVD mortality independently, or through interaction with weekly COVID-19 incidence.

#### Cancer

In 2020, we observed a significant negative association between weekly COVID-19 incidence and cancer mortality, while stringency of control measures showed a positive association with cancer mortality. In 2021, we found that stringency of control measures was negatively associated with cancer mortality, while a positive association was found between fully vaccinated individuals and cancer mortality**.**

## Discussion

Most countries revealed decreased cumulative mortality from respiratory disease and pneumonia, and excess cumulative mortality from CVD in 2020 and/or 2021. Our study also revealed distinct associations between excess mortality and COVID-19 incidence across time periods and different causes. Notably, stringent control measures were negatively associated with excess mortality from respiratory disease, CVD and cancer in 2021.

All countries analyzed reported decreased cumulative respiratory and pneumonia mortality, except for Georgia, Northern Ireland, Kazakhstan, and Ukraine, which exhibited excess respiratory and/or pneumonia mortality. Previous research has shown reduced non-COVID respiratory deaths [15] and declines in weekly pneumonia hospital admissions during the pandemic [16]. The decline in respiratory and pneumonia mortality in most countries may be attributed to the successful public health measures implemented to mitigate COVID-19 transmission, resulting also in reductions in common respiratory infections [17]. Contrarily, the surge in respiratory and pneumonia mortality observed in Kazakhstan and Ukraine could be explained by limited awareness and overwhelmed healthcare systems resulting in instances where patients passed away before receiving a formal COVID-19 diagnosis, leading to their classification as respiratory-related deaths [18, 19].

In line with the latter explanation, our study supports that mortality rates are influenced by the development level of each country. Specifically, upper- and lower-middle-income countries presented excess mortality from respiratory disease and pneumonia, whereas high-income countries presented decreased mortality from these causes. This suggests that higher-income countries had greater resourcefulness in their healthcare systems, exhibited better control of COVID-19 and other infections, and maintained accurate cause-of-death reporting, despite overwhelmed systems. Hence, the variation in respiratory mortality among countries is at least partly attributed to socioeconomic factors, mediated by healthcare access and quality disparities, as noted in other all-cause mortality studies [20].

Our findings of excess cumulative mortality from CVD, in 2020 and/or 2021, in all countries except Ukraine also align with previous research [21–23]. Reduced hospital admissions for myocardial infraction [24] and fewer prescriptions of life-saving cardiovascular medications [25] may indirectly contribute to excess CVD mortality. Pre-pandemic research also links social isolation, loneliness [26], and reduced daily activity to increased CVD risk. Lockdown measures inadvertently exacerbated these factors, underscoring the need for careful lockdown protocol decisions. On the other hand, Ukraine experienced decreased CVD, possibly because many COVID-19 patients with pre-existing CVD which increased their mortality risk were possibly documented as COVID-19 or respiratory deaths rather than CVD deaths [27].

Cyprus exhibited significant increases in cancer mortality percent change throughout all trimesters between 2015 and 2021. Contrarily, Brazil and Georgia presented decreases in all trimesters in 2020 and 2021, despite experiencing increases in cancer mortality in the previous trimesters. Limited research has explored cancer mortality during the COVID-19 pandemic. Previous research also demonstrated a decline in cancer mortality in Brazil during the pandemic compared to expected levels, however these findings did not reach statistical significance [27], while studies on disruptions in cancer care during this time predict a substantial increase in excess cancer mortality [28]. It is notable that countries experiencing reduced cancer mortality rates during 2020 and 2021 fall within the upper-middle income level. One plausible explanation for this trend could be potential inaccuracies in cause of death reporting during the pandemic years due to the stress of healthcare systems.

Surprisingly, in 2020, weekly reported COVID-19 cases were negatively associated with excess mortality from respiratory disease and cancer. In 2021, the same trend extended to pneumonia mortality. This is likely mediated by stringent mitigation measures in areas with high COVID-19 transmission, also reducing the spread of other respiratory infections, which could have indirectly mitigated cancer mortality as well [29]. In 2021, as many countries eased control measures, weekly incidence of COVID-19 was positively associated with respiratory disease, and CVD mortality, aligning with previous findings [30]. Additionally, we found that more stringent government measures had a protective effect on CVD mortality. Altogether, while COVID-19 incidence was positively associated with CVD deaths, CVD mortality was lower with stringent public health policies during 2021.

During 2020, a positive association was observed only between control measure stringency and cancer mortality. One explanation for this is that higher cancer mortality often correlates with older populations, prompting stricter control measures. Contrarily, the beneficial effect towards mortality from respiratory disease, CVD, and cancer, during 2021, endorses the effectiveness of control measures in mitigating excess mortality during the pandemic. Moreover, our findings are consistent with previous research, demonstrating that control measure stringency is associated with reduced all-cause excess mortality [10]. Stringency of control measures also decreased excess pneumonia and CVD mortality during 2020, through interaction with weekly reported COVID-19 cases; higher stringency measures in countries and weeks led to lower excess mortality compared to those with less stringent measures. However, during 2021, this interaction was associated with excess pneumonia, warranting further research to explore this.

This study also revealed a positive association between the percentage of fully vaccinated individuals and increased respiratory and cancer mortality. This is likely attributed to several factors, including prioritizing older and vulnerable populations in vaccine distribution. Additionally, the variability of the development level of participating countries could contribute to this; high-income countries tended to have greater vaccine access, encompassing larger proportions of older and at-risk populations. Conversely, lower- and middle-income countries experienced delays in achieving sufficient vaccination coverage compared to high-income countries. Therefore, substantial benefits may not have been reflected during 2021 [31].

Our results highlight the importance of effective and tailored public health interventions to mitigate pandemic-related mortality. Nevertheless, further research is warranted to fully grasp the intricate relationship between pandemic factors and mortality outcomes in each country’s context. More comprehensive risk–benefit analyses of public health interventions appear to be critical research areas for enhancing preparedness and response to future crises.

### Strengths and Limitations

This study is the first to analyse cause-specific excess mortality across multiple countries using national data sources for mortality. The method for estimating expected mortality demonstrated high accuracy compared to other methods, making it a reliable tool for estimating excess mortality [32]. Furthermore, by considering potential reporting delays, this study accurately represents each country’s mortality experience during 2020 and 2021. Lastly, this study is one of the few to investigate the impact of control measures on cause-specific mortality.

Our study, however, is not without limitations. As a country-level analysis, our findings indicate strong associations but not causation, necessitating further confirmation from larger cohorts and quasi-experimental designs. One significant constraint is the poor fit of cancer data in our model, preventing a comprehensive comparison of observed and expected mortality rates for this cause. Consequently, cancer results should be interpreted cautiously. Furthermore, the limited availability of accurate cause-specific mortality information worldwide, impedes the inclusion of more countries. Additionally, age-specific death rates were unavailable, preventing direct comparisons between countries. It is also worth noting that, while the World Health Organization’s ICD-10 codes provide a standardized system for cause-of-death recording, reporting practices differ significantly among countries. These discrepancies may stem from variations in how deaths are handled when multiple causes are involved or where medical information is limited, potentially leading to under- or over-reporting of deaths. Finally, the uncertainty around cause-of-death coding may be amplified in low- and middle-income countries due to limited healthcare infrastructure and less comprehensiveness of cause-of-death data in these regions [33].

## Conclusions

Our study across 12 countries and territories revealed significant declines in cumulative mortality rates from respiratory disease and pneumonia, along with excess cumulative mortality from CVD in most participating countries during 2020 and 2021. Our findings also support that public health interventions, such as stringency of control measures, mitigated the pandemic’s impact in terms of mortality from different causes.

### Supplementary Information

Below is the link to the electronic supplementary material.

Supplementary file1 (DOCX 4214 KB)

## Data Availability

The data and the associated statistical analysis codes underlying this study, beyond what is provided in the article and its online supplementary materials, can be made available upon request.

## Abbreviations

COVID-19: Coronavirus disease 2019
CVD: Cardiovascular disease
ICD-10: International Classification of Diseases, 10th Revision
C-MOR: COVID-19 MORtality Consortium
USA: United States of America
ISO: International Organization for Standardization
Epi week: Epidemiological week
UK: United Kingdom
CMRs: Crude mortality rates
GEE: Generalized estimating equation
GLM: Generalized linear model
