# Supplementary material for: Cause-Specific Excess Mortality During the COVID-19 Pandemic (2020–2021) in 12 Countries of the C-MOR Consortium
Source: J Epidemiol Glob Health. 2024 May 22;14(2):337–48. doi: 10.1007/s44197-024-00242-4 (PMC11176135; doi:10.1007/s44197-024-00242-4)
Supplement: Supplementary file 1 — Supplementary file1 (DOCX 4214 KB) [file 44197_2024_242_MOESM1_ESM.docx]

# SUPPLEMENTARY MATERIAL

**Table S1. National data sources, period of available mortality data, time unit, data quality of civil registration and vital statistics systems, and income level per country**

| **Country** | **Partners** | **Access Date** | **Source** | **Time Unit** | **% Completeness of vital registration systems*** | **Income Level** | **Economic income (2022 US$)** |
| --- | --- | --- | --- | --- | --- | --- | --- |
| Australia | Deakin University | May 9th, 2022 | Australian Bureau of Statistics | ISO | 100 | High | 1.68 trillion |
| Austria | Department for Epidemiology, Center for Public Health, Medical University of Vienna | August 22^nd^, 2022 | Official Cause-of-death statistics, Statistics Austria | ISO | 100 | High | 471.4 billion |
| Brazil | Federal University of Rio de Janeiro and Fluminense Federal University | July 13^th^, 2022 | Datasus, Ministry of Health Brazil | Epi | 99.3 | Upper-middle | 1.92 trillion |
| Cyprus | University of Nicosia & Health Monitoring Unit, Cyprus Ministry of Health | June 2022 | Eurostat | ISO | 90.7 | High | 28.44 billion |
| England and Wales | St George's, University of London | June 25th, 2022 | Office for National Statistics | National | 100 | High | 3.07 trillion |
| Georgia | National Center for Disease control and Public Health (Primary organization), National Statistics Office of Georgia (partner organization) | June 2021, June 2022 | Vital Registration System | ISO | 94.3 | Upper-middle | 24.61 billion |
| Kazakhstan | Asfendiyarov Kazakh National Medical University | 2021-2022 | Ministry of health reports and the Republican Center of e-health records | ISO | 88.3 | Upper-middle | 220.62 billion |
| Northern Ireland | St George's, University of London | June 25th, 2022 | Northern Ireland Statistics and Research Agency | National | 100 | High | 3.07 trillion |
| Scotland | St George's, University of London | June 25th, 2022 | National Records of Scotland | ISO | 100 | High | 3.07 trillion |
| Slovenia | National Institute of Public Health | 2022 | Human Mortality Database | ISO | 94.8 | High | 62.12 billion |
| Ukraine | Bogomolets National Medical University | January 2022 | Bogomolets National medical University | ISO | 100 | Lower-middle | 160.5 billion |
| USA | Our World in Data | July 2022 | Human Mortality Database (HMD) and the World Mortality Dataset (WMD) | Epi | 99.9 | High | 25.46 trillion |

*For Scotland, Northern Ireland, England and Wales, income level from the UK was used.*

*Non-shaded area indicates countries of high-income level.*

*Light-shaded area indicates countries of upper-middle income level.*

*Dark-shaded area indicates countries of low-middle income level.*

**Table S2. Publicly available sources for the database of country-level pandemic related variables (reported weekly)**

| **Variable Label** | **Description** | **Time unit** | **Time span** | **Data Source** | **Weblink** |
| --- | --- | --- | --- | --- | --- |
| Country | Country | NA | - | - | - |
| Year | Report year | NA | - | - | - |
| Week | Report week | NA | - | - | - |
| Population | Annual country population (July 1st) | Year | 2020-2021 | Department of Economic and Social Affairs, Population Division, United Nation | [population](https://population.un.org/wpp/Download/Standard/Population/) |
| Stringency index | Mean stringency index per week | Week | 2020-2021 | Blavatnik School of Government, University of Oxford | [stringency_index](https://ourworldindata.org/explorers/coronavirus-data-explorer?uniformYAxis=0&hideControls=true&Interval=7-day+rolling+average&Relative+to+Population=true&Color+by+test+positivity=false&country=USA~ITA~CAN~DEU~GBR~FRA&Metric=Stringency+index) |
| Fully vaccinated | People fully vaccinated per hundred per week | Week | 2020-2021 | Our World in Data | [fully_vaccinated](https://ourworldindata.org/covid-vaccinations) |
| COVID-19 reported incidence | Weekly incidence of reported COVID-19 cases per 100,000 population | Week | 2020-2021 | Our World in Data | [COVID-19 incidence](https://ourworldindata.org/covid-models#institute-for-health-metrics-and-evaluation-ihme) |

*For Scotland, Northern Ireland, England and Wales, data is only available for "population" and "vaccination". Hence, for other variables, data from the UK was used.*

**Table S3. Truncated weeks excluded from the Generalized Linear Model by cause and country**

| **Cause** | **Country** | **Truncated Weeks** |
| --- | --- | --- |
| **Respiratory Disease** | Australia, Austria, Brazil, Cyprus, Georgia, Northern Ireland, Kazakhstan, USA, Scotland | Week 53 |
|  | Slovenia | Week 52 |
|  | England and Wales | Week 1, Week 52 |
| **Pneumonia** | Austria, Australia, Brazil, Georgia, Cyprus, Northern Ireland, Slovenia, Ukraine, Kazakhstan | Week 53 |
| **Cardiovascular Disease** | Austria, Australia, Brazil, Cyprus, Georgia, Northern Ireland, Slovenia and the USA | Week 53 |

**
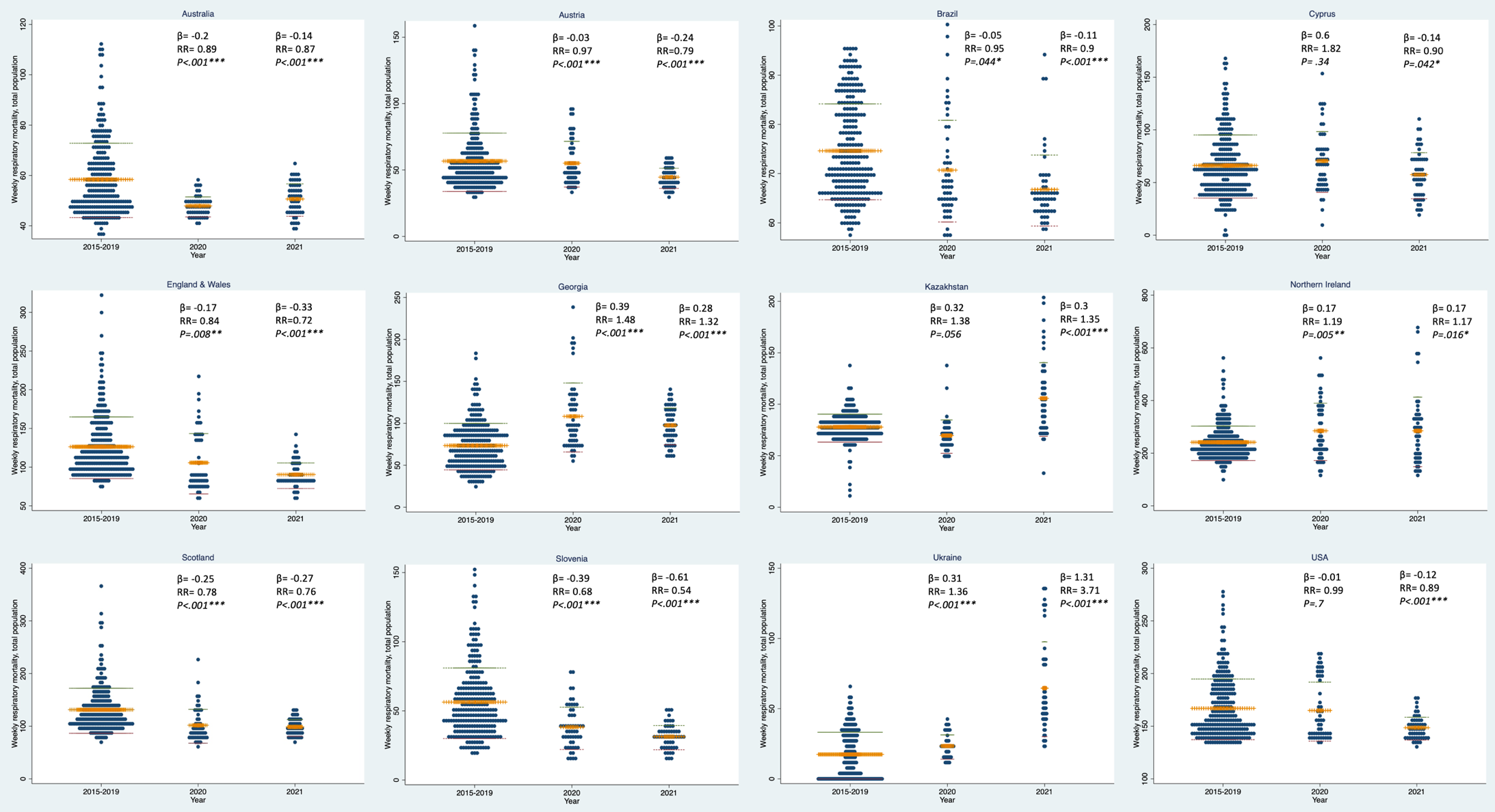
**

**Figure S1. Weekly mortality from respiratory disease for the total population 2020 and 2021 compared to 2015-2019 in 12 countries of the C-MOR consortium**

*The figure demonstrates the average weekly mortality rates from respiratory disease for the total population (y-axis) in 2015-2019, 2020 and 2021 (x-axis).*

*β coefficients and risk ratios compare each of 2020 and 2021 weekly average mortality rates (per 100,000 population) to the baseline (2015-2019) from the GEE analysis.*

*​•= number of deaths per 100,000 population for a particular week​, +++= weekly average for each time period, ---/---= mean +/- SD.*

*p<.001***, p<.01**, p<.05*.*

**Figure S1 description:** The GEE regression coefficients suggest that compared to baseline mortality rates, respiratory disease mortality significantly decreased in both 2020 and 2021 in Australia, Austria, Brazil, England and Wales, Scotland, and Slovenia. In Cyprus and the USA, mortality rates from respiratory disease significantly decreased only in 2021, compared to the baseline period of 2015-2019. In Georgia, Northern Ireland and Ukraine, mortality rates from respiratory disease significantly increased in both years, whereas in Kazakhstan, mortality rates from respiratory disease significantly increased only in 2021.


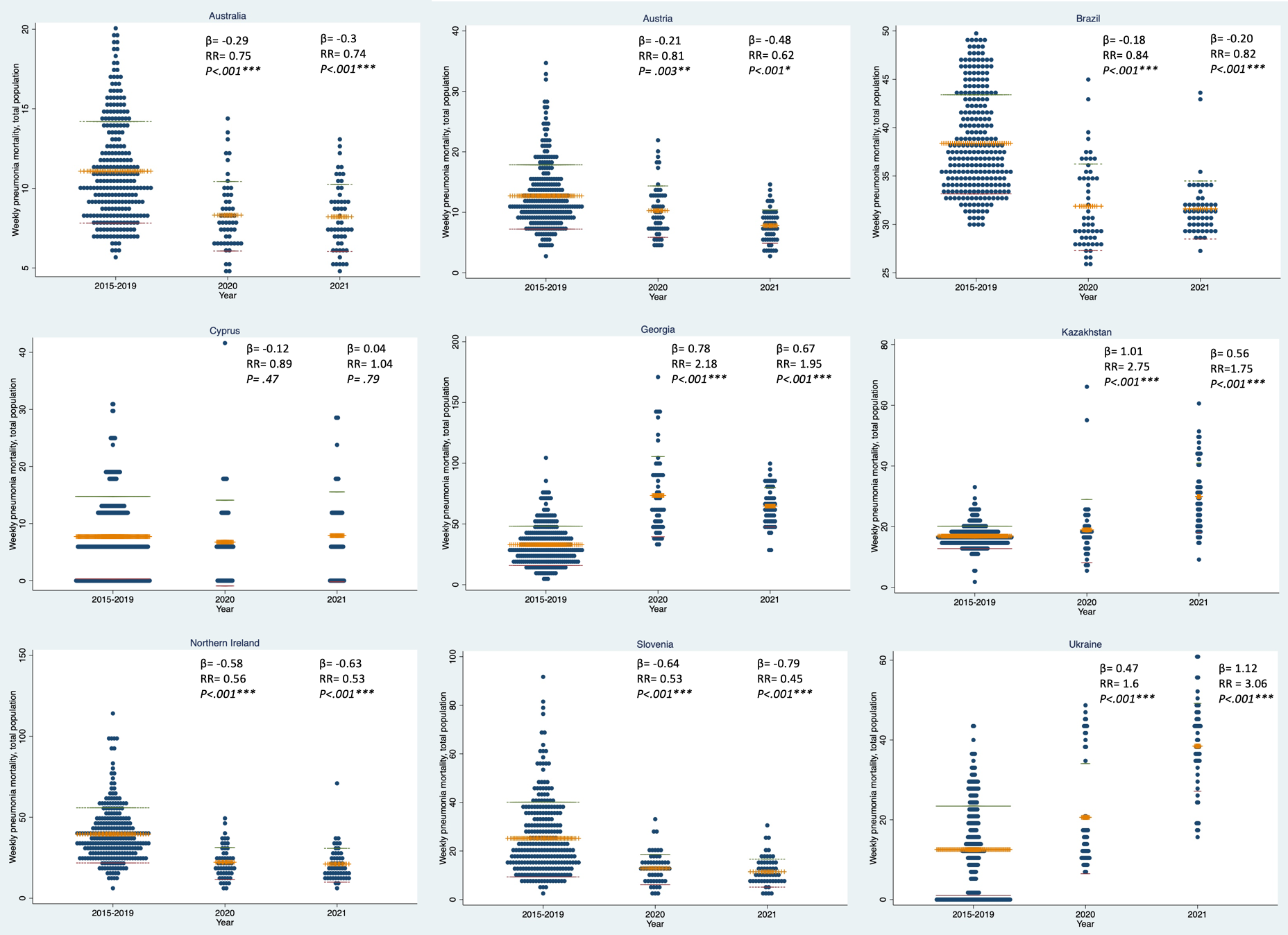


**Figure S2. Weekly mortality from pneumonia for the total population 2020 and 2021 compared to 2015-2019 in 9 countries of the C-MOR consortium**​

*The figure demonstrates the average weekly mortality rates from pneumonia for the total population (y-axis) in 2015-2019, 2020 and 2021 (x-axis).*

*β coefficients and risk ratios compare each of 2020 and 2021 weekly average mortality rates (per 100,000 population) to the baseline (2015-2019) from the GEE analysis.*

​*•= number of deaths per 100,000 population for a particular week​, +++= weekly average for each time period, ---/---= mean +/- SD.*

*p<.001***, p<.01**, p<.05*.*

**Figure S2 description:** The GEE regression coefficients suggest that pneumonia mortality rates decreased significantly in Australia, Austria, Brazil, Northern Ireland, and Slovenia, and increased significantly in Georgia, Kazakhstan, and Ukraine, during both 2020 and 2021, compared to baseline.

**
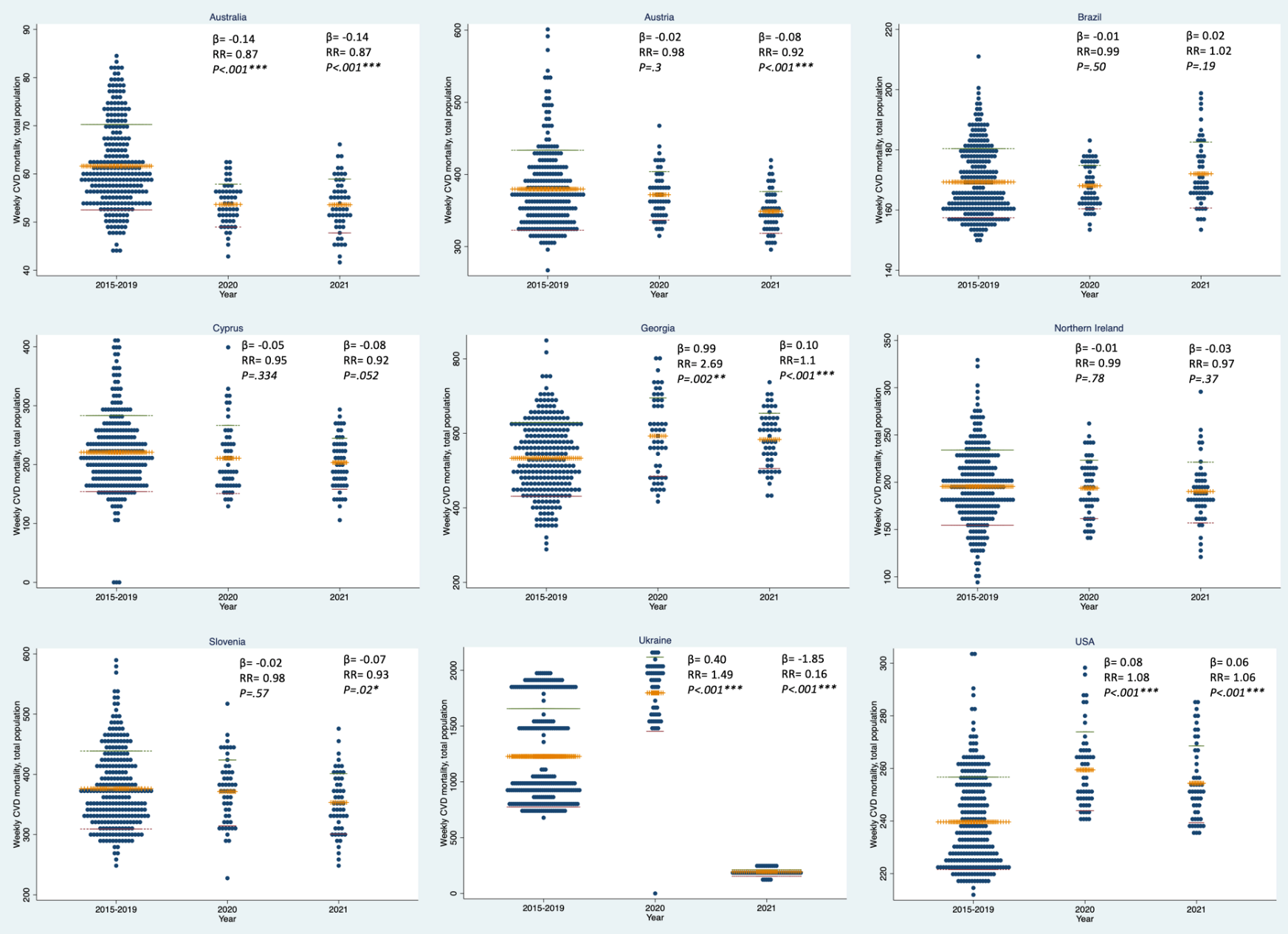
**

**Figure S3. Weekly mortality from cardiovascular disease for the total population 2020 and 2021 compared to 2015-2019 in 9 countries of the C-MOR consortium**

*The figure demonstrates the average weekly mortality rates from cardiovascular disease for the total population (y-axis) in 2015-2019, 2020 and 2021 (x-axis).*

*β coefficients and risk ratios compare each of 2020 and 2021 weekly average mortality rates (per 100,000 population) to the baseline (2015-2019) from the GEE analysis.*

​•= number of deaths per 100,000 population for a particular week​, +++= weekly average for each time period, ---/---= mean +/- SD

*p<.001***, p<.01**, p<.05*.*

**Figure S3 Description:** The GEE coefficients demonstrated significant increases in mortality rates from cardiovascular disease in Georgia and the USA, while decreases were observed in Australia in both 2020 and 2021, and in Austria and Slovenia in 2021, compared to the baseline period. Ukraine experienced significant increases in mortality rates from CVD, in 2020, but significant decreases in 2021, compared to baseline.


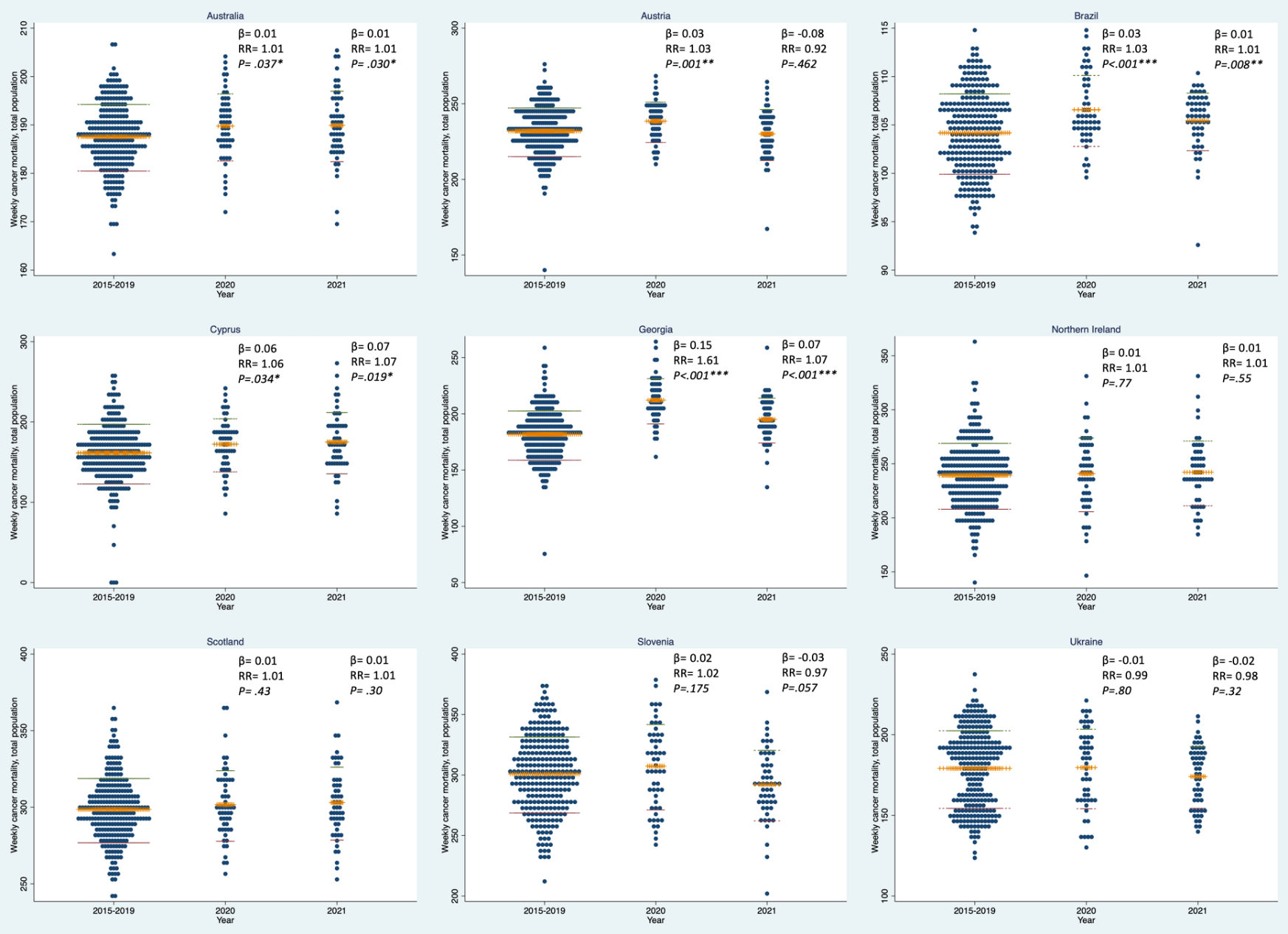


**Figure S4. Weekly mortality from cancer, for the total population, in 2015-2019, 2020 and 2021 in 9 countries of the C-MOR consortium**​

*The figure demonstrates the average weekly mortality rates from cancer for the total population (y-axis) in 2015-2019, 2020 and 2021 (x-axis).*

*β coefficients and risk ratios compare each of 2020 and 2021 weekly average mortality rates (per 100,000 population) to the baseline (2015-2019) from the GEE analysis.*

​•= number of deaths per 100,000 population for a particular week​, +++= weekly average for each time period, ---/---= mean +/- SD

*p<.001***, p<.01**, p<.05*.*

**Figure S4 Description:** The GEE coefficients demonstrated significant increases in observed weekly mortality rates due to cancer during both years in Australia, Brazil, Cyprus and Georgia, compared to baseline mortality rates. Austria experienced significant increases in mortality rates from cancer only in 2020, while the rest of the countries did not experience any significant changes, compared to baseline.

**Table S4. Weeks with substantial increase/decrease of mortality from respiratory disease, pneumonia and cardiovascular disease in 2020 and/or 2021 from countries of the C-MOR consortium**

|  | **Respiratory Disease** | | **Pneumonia** | | **Cardiovascular disease** | |
| --- | --- | --- | --- | --- | --- | --- |
| **Country** | **2020 Week** | **2021 Week** | **2020 Week** | **2021 Week** | **2020 Week** | **2021 Week** |
| Austria |  | 🡫10 |  |  |  |  |
| Cyprus | 🡩21 |  |  |  | 🡩21 |  |
| Georgia |  | 🡫 52 | 🡩46-50 |  |  |  |
| Kazakhstan | 🡩25-31 | 🡫1 🡩28, 30-46, 48 | 🡩25-31 | 🡩30-45 |  |  |
| Northern Ireland | 🡩14-17, 19, 44-48 | 🡩1-5, 35, 37-39 |  |  |  |  |
| Slovenia |  | 🡫4, 6-9 |  |  |  | 🡫52 |
| Ukraine | 🡩12-39 | 🡩4, 8-52 |  | 🡩15-32, 34 |  | 🡫1-52 |
| USA |  | 🡫6, 8-9 |  |  |  |  |

*↑ Indicates substantial increase in excess mortality (z-score>4).*

*↓ Indicates substantial decrease (z-score<-4).*

**Table S4 description:** *Respiratory Disease;* The countries that experienced substantial excess respiratory mortality (z-score> 4) in any week during 2020 and 2021 include Cyprus (2020; 1 week), Kazakhstan (2020; 7 weeks, 2021; 19 weeks), Northern Ireland (2020; 10 weeks, 2021; 9 weeks) and Ukraine (2020; 27 weeks, 2021; 46 weeks). Austria (1 week), Georgia (1 week), Kazakhstan (1 week) and the USA (3 weeks), experienced a substantial decrease (z-score< -4) in respiratory mortality rates during 2021, while no substantial decreases were demonstrated during the year 2020.

*Pneumonia*; Kazakhstan experienced substantial excess (z-score> 4) mortality rates from pneumonia during both years (2020; 7 weeks, 2021; 16 weeks). Substantial excess pneumonia mortality was also demonstrated in Georgia (5 weeks) during 2020, and Ukraine (19 weeks) during 2021, while no other substantial changes were noticed in the other participating countries.

*Cardiovascular Disease;* There were no notable changes in weekly CVD mortality rates in all participating countries, except for Cyprus, which experienced excess mortality in 2020 (1 week), and Slovenia (1 week) and Ukraine (throughout all weeks), which experienced substantial decrease (z-score< -4) in 2021.


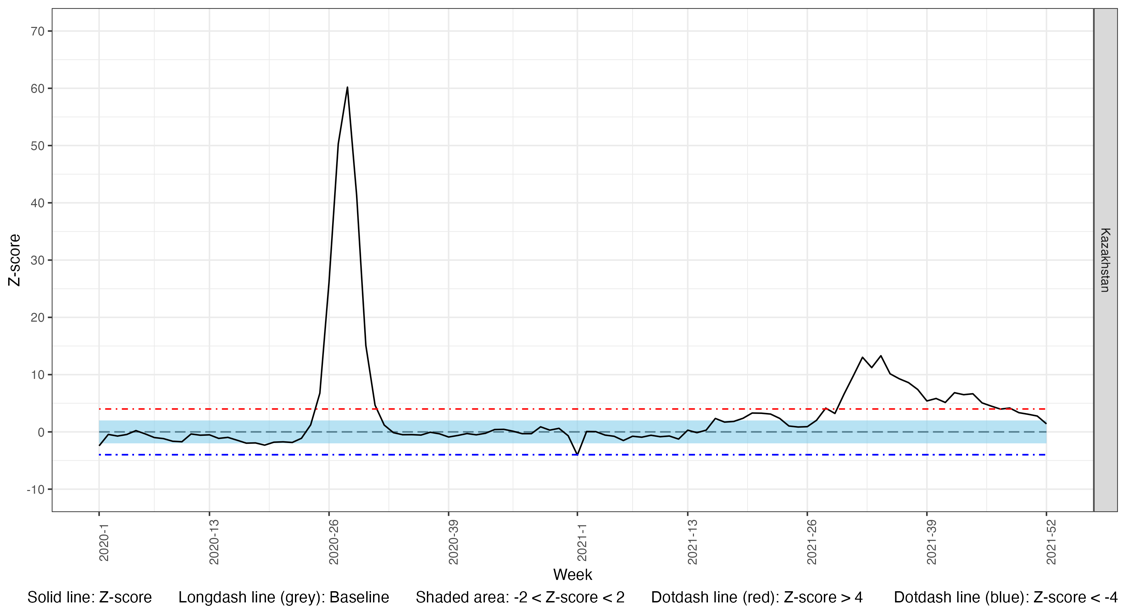


**Figure S5. Weekly z-score of mortality rates from respiratory disease for the total population of Kazakhstan in 2020 and 2021**


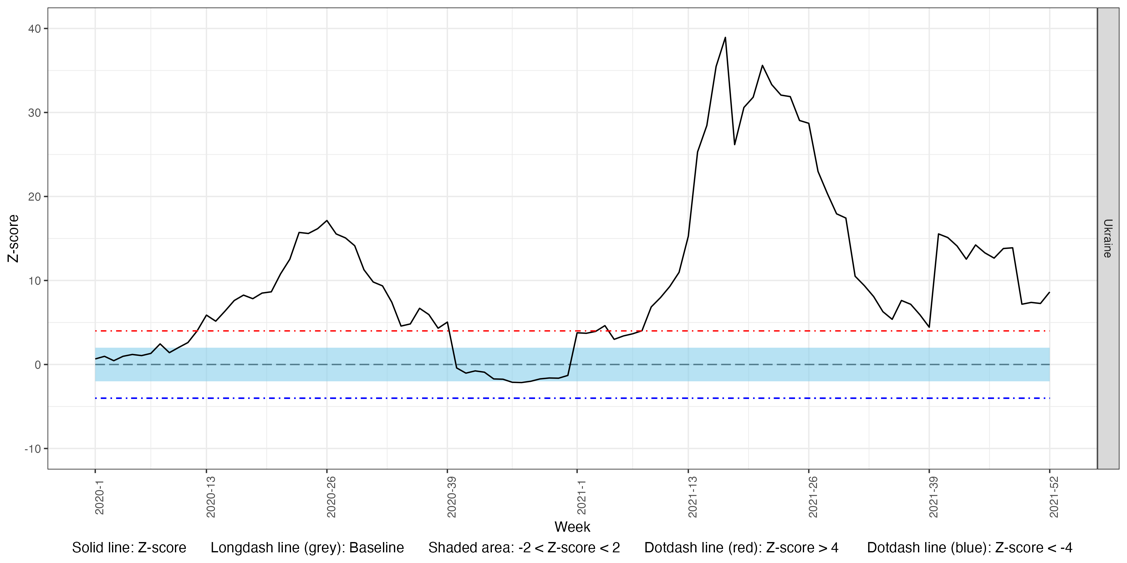


**Figure S6. Weekly z-score of mortality rates from respiratory disease for the total population of Ukraine in 2020 and 2021**

**
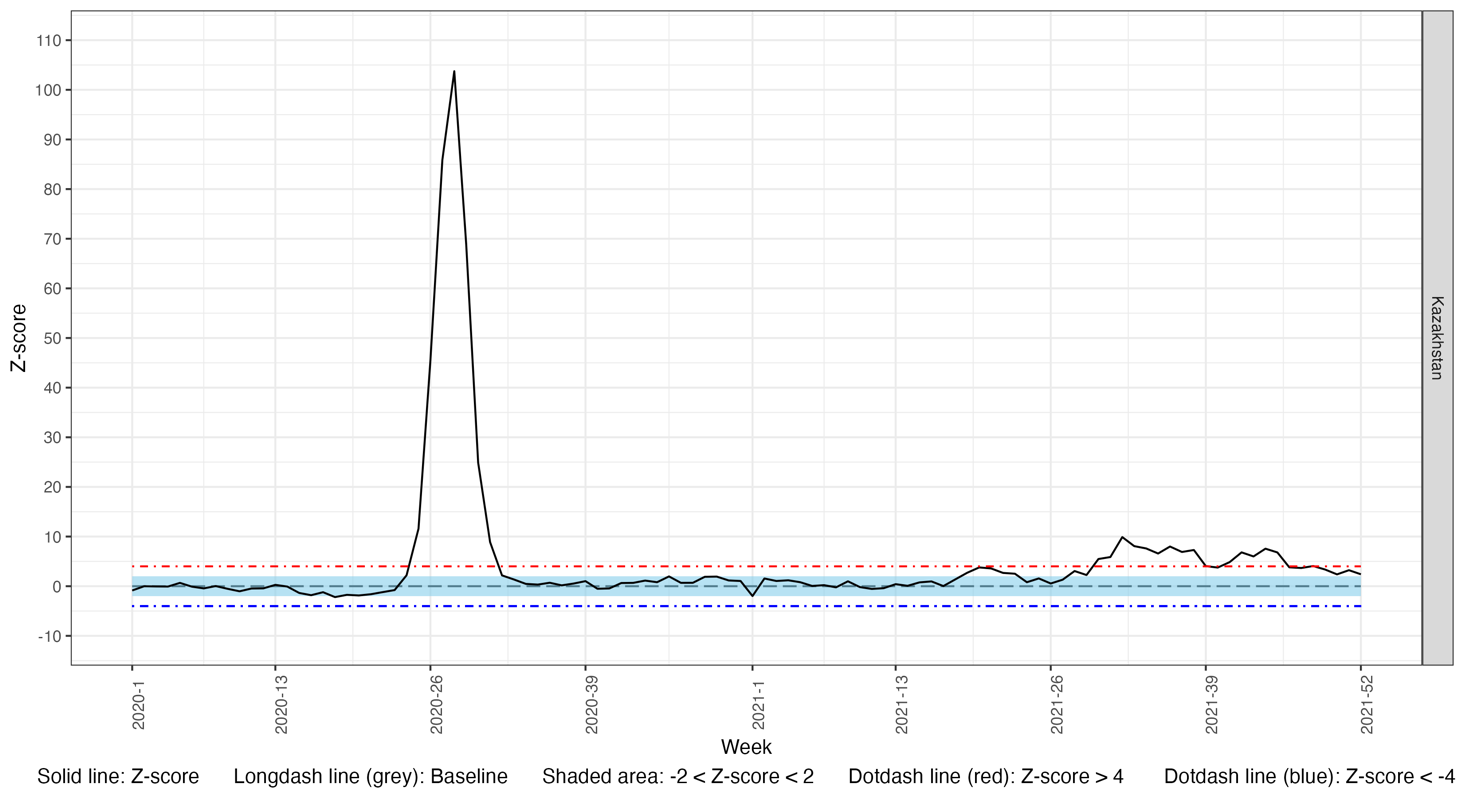
**

**Figure S7. Weekly z-score of mortality rates from pneumonia for the total population of Kazakhstan in 2020 and 2021**

**Table S5. Comparison of the cumulative observed and expected mortality rate from respiratory disease per 100,000 population for the whole year 2020 and 2021 in 12 countries of the C-MOR consortium**

| **Year** | **2020** | | | | | | | **2021** | | | | | | |
| --- | --- | --- | --- | --- | --- | --- | --- | --- | --- | --- | --- | --- | --- | --- |
| **Country** | **Expected mortality rate** | **Observed mortality rate** | **Lower limit of 95% CI of Expected mortality rate** | **Upper limit of 95% CI of Expected mortality rate** | **Difference (Observed-Expected mortality rate)** | **Difference using the lower limit of 95% CI of Expected mortality rate** | **Difference using the upper limit of 95% CI of Expected mortality rate** | **Expected mortality rate** | **Observed mortality rate** | **Lower limit of 95% CI of Expected mortality rate** | **Upper limit of 95% CI of Expected mortality rate** | **Difference (Observed-Expected mortality rate)** | **Difference using the lower limit of 95% CI of Expected mortality rate** | **Difference using the upper limit of 95% CI of Expected mortality rate** |
| **Australia** | 3078.697 | 2490.19 | 57.08805 | 61.34892 | -11.3174 🡫 | -13.4607 | -9.19978 | 3092.07 | 2633.192 | 57.32594 | 61.62573 | -8.82458 🡫 | -10.9874 | -6.68763 |
| **Austria** | 3389.367 | 2871.996 | 61.72911 | 68.6932 | -9.94945 🡫 | -13.4625 | -6.49841 | 3555.335 | 2321.386 | 64.80389 | 72.00294 | -23.7298 🡫 | -27.3609 | -20.1618 |
| **Brazil** | 3956.241 | 3682.42 | 73.67978 | 78.5089 | -5.26579 🡫 | -7.69313 | -2.864 | 3982.291 | 3472.34 | 74.1563 | 79.03465 | -9.80675 🡫 | -12.2589 | -7.38053 |
| **Cyprus** | 3898.248 | 3696.849 | 68.55913 | 81.5615 | -3.87306 | -10.4683 | 2.534113 | 4048.446 | 3006.227 | 71.26719 | 84.63358 | -20.0427 🡫 | -26.8215 | -13.4551 |
| **England & Wales** | 5917.504 | 5274.998 | 109.3942 | 118.2597 | -12.3559 🡫 | -16.8174 | -7.95192 | 5790.319 | 4500.302 | 106.9684 | 115.7945 | -24.808 🡫 | -29.2502 | -20.4241 |
| **Georgia** | 5829.888 | 5622.41 | 106.3206 | 118.0074 | -3.98997 | -9.88415 | 1.802666 | 6781.659 | 5076.876 | 124.0699 | 136.8679 | -32.7843 🡫 | -39.2356 | -26.4376 |
| **Kazakhstan** | 3802.428 | 5614.196 | 70.38054 | 75.90143 | 34.8417 🡩 | 32.06389 | 37.58478 | 3717.1 | 5496.762 | 68.75604 | 74.24446 | 34.22427 🡩 | 31.4625 | 36.95092 |
| **N. Ireland** | 11597.27 | 14816.7 | 212.3831 | 233.8377 | 61.91211 🡩 | 51.09876 | 72.55345 | 11312.01 | 14813.95 | 206.972 | 228.2793 | 67.34507 🡩 | 56.60448 | 77.91171 |
| **Scotland** | 5878.635 | 5300.97 | 107.6288 | 118.5606 | -11.1089 🡫 | -16.6189 | -5.68706 | 5589.463 | 5128.65 | 102.1771 | 112.8912 | -8.8618 🡫 | -14.2634 | -3.54925 |
| **Slovenia** | 2371.361 | 1967.189 | 41.7016 | 49.61922 | -7.77255 🡫 | -11.7887 | -3.87104 | 2205.945 | 1609.505 | 38.64323 | 46.31654 | -11.47 🡫 | -15.3645 | -7.69122 |
| **Ukraine** | 688.9197 | 1228.267 | 11.87927 | 14.66654 | 10.37207 🡩 | 8.953981 | 11.74125 | 629.1299 | 3358.87 | 10.78268 | 13.46419 | 52.49501 🡩 | 51.12947 | 53.81098 |
| **USA** | 8628.878 | 8570.191 | 162.3878 | 169.5177 | -1.12858 | -4.70631 | 2.423615 | 8609.889 | 7727.949 | 162.003 | 169.1725 | -16.9604 🡫 | -20.5581 | -13.3886 |

*↑ Indicates statistically significant excess respiratory mortality using the sum of deaths for 2020 and 2021.*

*↓ Indicates a statistically significant reduction respiratory mortality using the sum of deaths for 2020 and 2021.*

*Non-shaded area indicates countries of high-income level.*

*Light-shaded area indicates countries of upper-middle income level.*

*Dark-shaded area indicates countries of low-middle income level.*

**Table S6. Comparison of the cumulative observed and expected mortality rate from pneumonia per 100,000 population for the whole year 2020 and 2021 in 9 countries of the C-MOR consortium**

| **Year** | **2020** | | | | | | | **2021** | | | | | | |
| --- | --- | --- | --- | --- | --- | --- | --- | --- | --- | --- | --- | --- | --- | --- |
| **Country** | **Expected mortality rate** | **Observed mortality rate** | **Lower limit of 95% CI of Expected mortality rate** | **Upper limit of 95% CI of Expected mortality rate** | **Difference (Observed-Expected mortality rate)** | **Difference using the lower limit of 95% CI of Expected mortality rate** | **Difference using the upper limit of 95% CI of Expected mortality rate** | **Expected mortality rate** | **Observed mortality rate** | **Lower limit of 95% CI of Expected mortality rate** | **Upper limit of 95% CI of Expected mortality rate** | **Difference (Observed-Expected mortality rate)** | **Difference using the lower limit of 95% CI of Expected mortality rate** | **Difference using the upper limit of 95% CI of Expected mortality rate** |
| **Australia** | 586.5666 | 434.6115 | 10.36293 | 12.22289 | -2.92221 🡫 | -3.86497 | -2.00502 | 590.1405 | 427.0963 | 10.42241 | 12.30123 | -3.13547 🡫 | -4.08784 | -2.20902 |
| **Austria** | 779.6763 | 534.2972 | 13.88371 | 16.13195 | -4.71883 🡫 | -5.857 | -3.60876 | 825.4307 | 405.6452 | 14.72013 | 17.05586 | -8.0728 🡫 | -9.25499 | -6.91926 |
| **Brazil** | 2012.507 | 1660.828 | 36.99301 | 40.43665 | -6.76306 🡫 | -8.49765 | -5.05401 | 2018.083 | 1643.222 | 37.08642 | 40.55804 | -7.20887 🡫 | -8.95762 | -5.486 |
| **Cyprus** | 385.1876 | 340.2008 | 5.454239 | 9.550373 | -0.86513 🡫 | -3.00805 | 1.088084 | 377.9994 | 417.8539 | 5.323838 | 9.406692 | 0.766431 | -1.37104 | 2.711814 |
| **Georgia** | 2707.221 | 3752.026 | 48.21987 | 56.00095 | 20.09241 🡩 | 16.1534 | 23.93447 | 3192.157 | 3363.828 | 57.14244 | 65.73304 | 3.301372 | -1.04403 | 7.546566 |
| **Kazakhstan** | 865.8095 | 2429.96 | 15.53413 | 17.79176 | 30.07982 🡩 | 28.93824 | 31.19587 | 860.0425 | 1557.578 | 15.42033 | 17.68405 | 13.41415 🡩 | 12.26938 | 14.5331 |
| **N. Ireland** | 1731.858 | 1154.94 | 30.39659 | 36.30058 | -11.0946 🡫 | -14.0902 | -8.18621 | 1640.225 | 1097.33 | 28.70035 | 34.47332 | -10.4403 🡫 | -13.3708 | -7.59785 |
| **Slovenia** | 750.0809 | 673.2491 | 12.48579 | 16.45458 | -1.47753 🡫 | -3.50748 | 0.461306 | 626.1608 | 596.2036 | 10.27062 | 13.90398 | -0.5761 | -2.43852 | 1.194831 |
| **Ukraine** | 775.9116 | 1046.61 | 12.83553 | 17.10937 | 5.20574 🡩 | 3.017744 | 7.29159 | 826.1119 | 2000.128 | 13.70258 | 18.17606 | 22.57723 🡩 | 20.28793 | 24.76142 |

*↑ Indicates statistically significant excess respiratory mortality using the sum of deaths for 2020 and 2021.*

*↓ Indicates a statistically significant reduction respiratory mortality using the sum of deaths for 2020 and 2021.*

*Non-shaded area indicates countries of high-income level.*

*Light-shaded area indicates countries of upper-middle income level.*

*Dark-shaded area indicates countries of low-middle income level.*

**Table S7. Comparison of the cumulative observed and expected mortality rate from CVD per 100,000 population for the whole year 2020 and 2021 in 9 countries of the C-MOR consortium**

| **Year** | **2020** | | | | | | | **2021** | | | | | | | |
| --- | --- | --- | --- | --- | --- | --- | --- | --- | --- | --- | --- | --- | --- | --- | --- |
| **Country** | **Expected mortality rate** | **Observed mortality rate** | **Lower limit of 95% CI of Expected mortality rate** | **Upper limit of 95% CI of Expected mortality rate** | **Difference (Observed-Expected mortality rate)** | **Difference using the lower limit of 95% CI of Expected mortality rate** | **Difference using the upper limit of 95% CI of Expected mortality rate** | **Expected mortality rate** | **Observed mortality rate** | **Lower limit of 95% CI of Expected mortality rate** | **Upper limit of 95% CI of Expected mortality rate** | **Difference (Observed-Expected mortality rate)** | **Difference using the lower limit of 95% CI of Expected mortality rate** | **Difference using the upper limit of 95% CI of Expected mortality rate** |  |
| **Australia** | 2730.057 | 2792.456 | 50.51293 | 54.51466 | 1.199998 | -0.81358 | 3.188154 | 2588.256 | 2787.059 | 47.82963 | 51.74434 | 3.823129 🡩 | 1.852946 | 5.767651 |  |
| **Austria** | 18002.2 | 19330.49 | 337.8402 | 354.6199 | 25.54406 🡩 | 17.1203 | 33.90004 | 17451.94 | 18144.46 | 327.3427 | 343.9542 | 13.31778 🡩 | 4.977763 | 21.58928 |  |
| **Brazil** | 8786.531 | 8743.486 | 165.3876 | 172.5814 | -0.82779 | -4.43747 | 2.756359 | 8780.619 | 8946.254 | 165.2518 | 172.4902 | 3.185276 | -0.44686 | 6.791556 |  |
| **Cyprus** | 10189.42 | 10999.83 | 185.3595 | 206.7358 | 15.58463 🡩 | 4.799334 | 26.17556 | 9750.62 | 10672.68 | 177.1018 | 198.1184 | 17.73201 🡩 | 7.125564 | 28.14212 |  |
| **Georgia** | 29339.19 | 30629.82 | 547.4853 | 581.1121 | 24.8198 🡩 | 7.922907 | 41.54968 | 29910.39 | 30350.56 | 558.1815 | 592.3878 | 8.464769 | -8.72314 | 25.48315 |  |
| **N. Ireland** | 9467.014 | 10073.49 | 172.805 | 191.4704 | 11.66299 🡩 | 2.250554 | 20.91592 | 9250.769 | 9892.43 | 168.7038 | 187.2562 | 12.33963 🡩 | 2.982791 | 21.53521 |  |
| **Slovenia** | 19059.15 | 19303.98 | 355.72 | 377.4315 | 4.708269 | -6.20107 | 15.51043 | 18869.71 | 18369.45 | 352.0638 | 373.8027 | -9.62026 | -20.544 | 1.194926 |  |
| **Ukraine** | 112917.7 | 95240.19 | 2077.919 | 2266.433 | -339.952 🡫 | -434.891 | -246.377 | 138863.2 | 10058.71 | 2564.625 | 2777.684 | -2477.01 🡫 | -2584.25 | -2371.19 |  |
| **USA** | 12666.99 | 13454.68 | 239.2888 | 247.9285 | 15.14807 | 10.81544 | 19.45516 | 12737.19 | 13227.8 | 240.5976 | 249.3204 | 9.434672 | 5.060373 | 13.78308 |  |

*↑ Indicates statistically significant excess mortality from cardiovascular disease using the sum of deaths for 2020 and 2021.*

*↓ Indicates a statistically significant reduction in mortality from cardiovascular disease using the sum of deaths for 2020 and 2021.*

*Non-shaded area indicates countries of high-income level.*

*Light-shaded area indicates countries of upper-middle.*

*Dark-shaded area indicates countries of low-middle income level.*

**
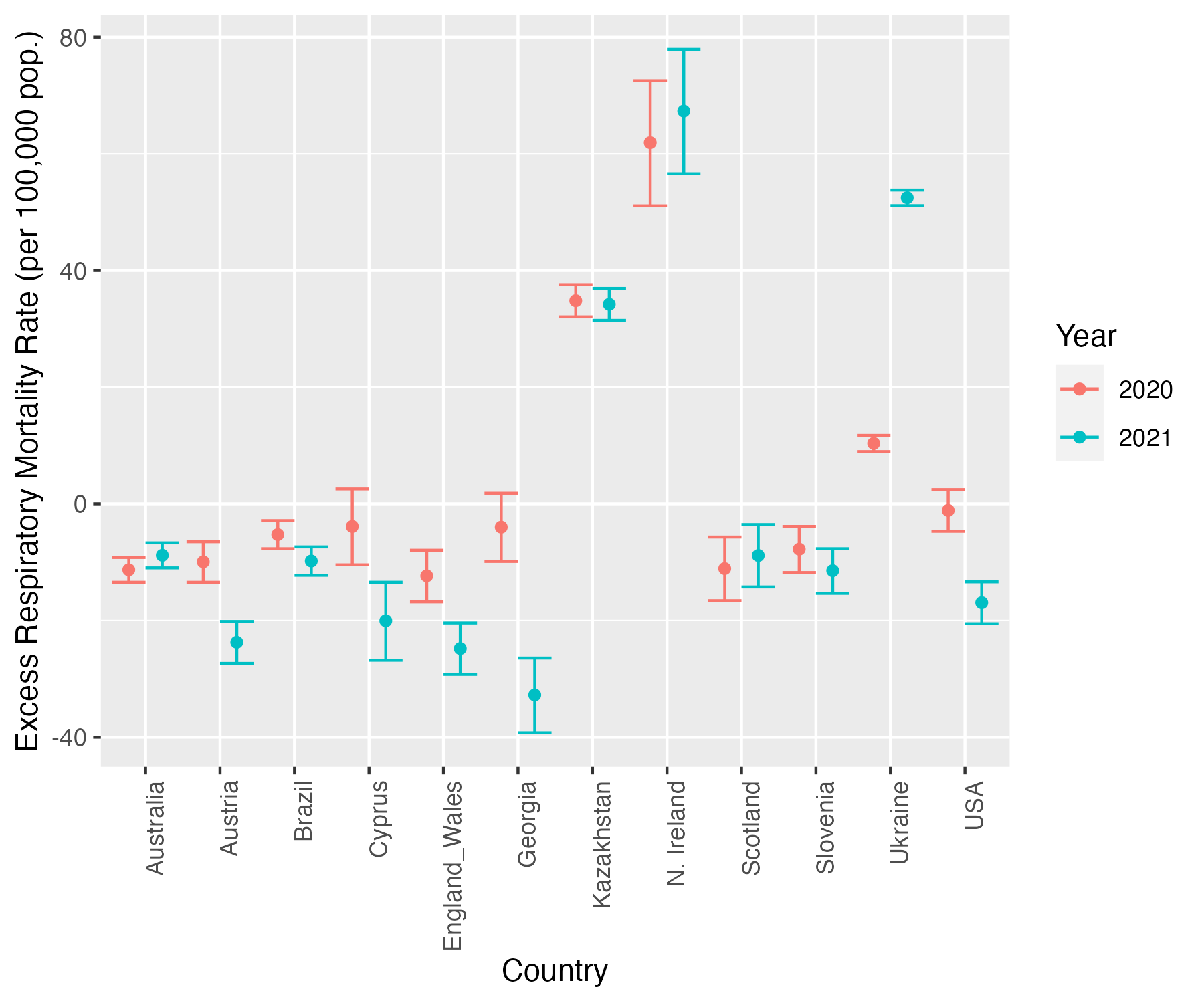
**

**Figure S8. Cumulative excess mortality rate from respiratory disease for 2020 and 2021 in 12 countries of the C-MOR consortium**

**
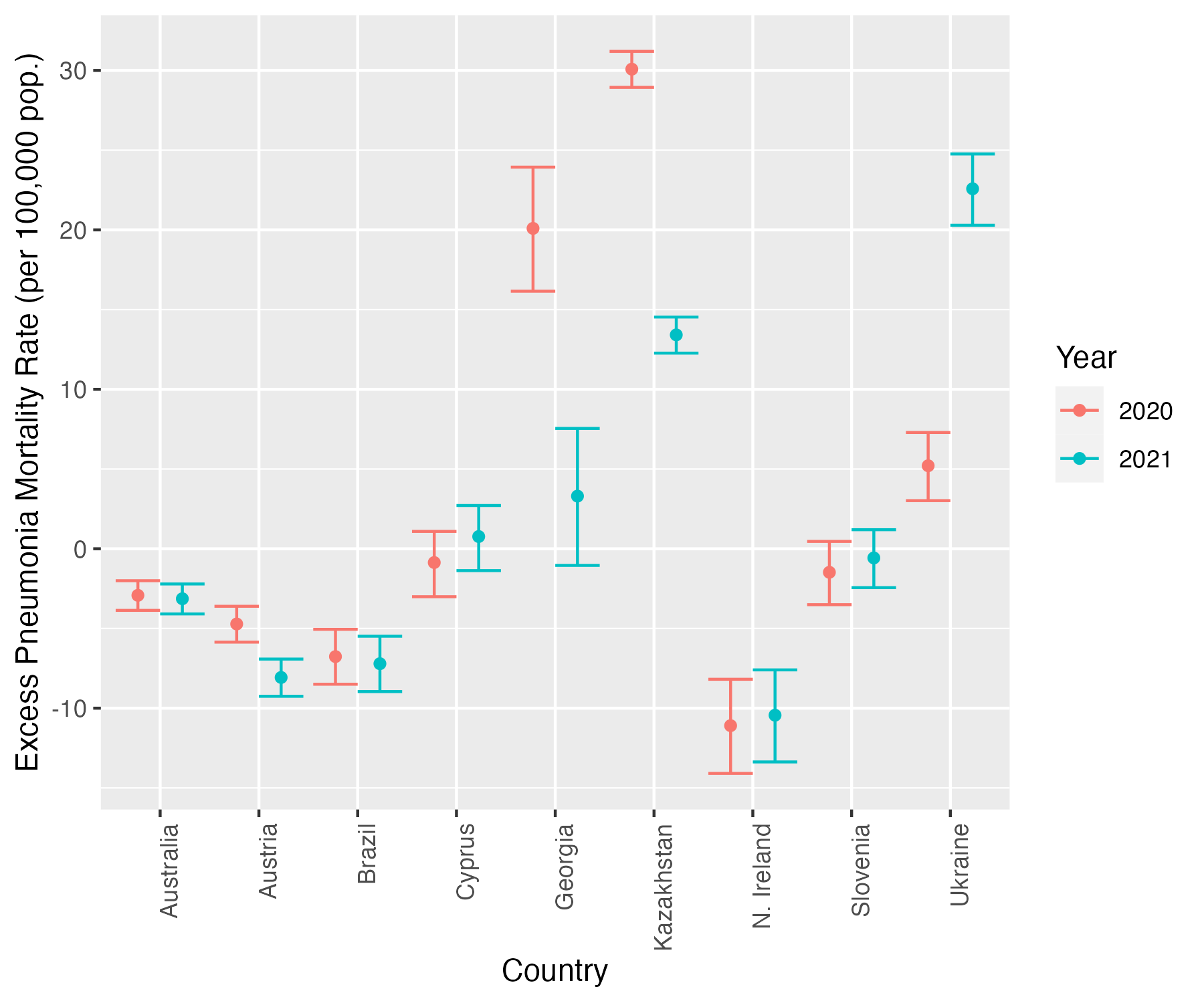
**

**Figure S9. Cumulative excess mortality rate from pneumonia for 2020 and 2021 in 9 countries of the C-MOR consortium**

**
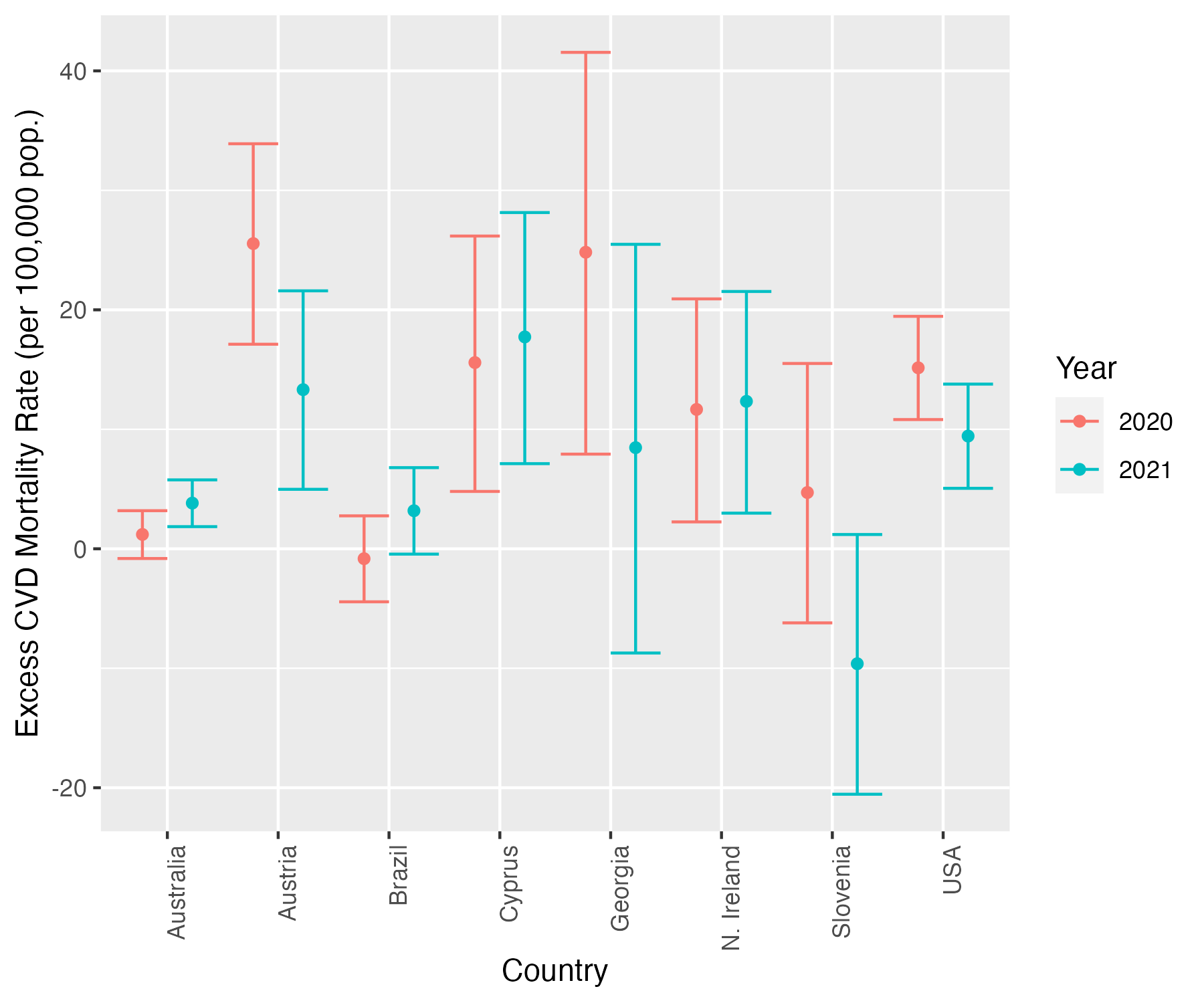
**

**Figure S10. Cumulative excess mortality rate from CVD for 2020 and 2021 in 8 countries of the C-MOR consortium**

*To ensure a clear and concise figure Ukraine was omitted from the above plot.*

**Table S8.** **Cumulative crude cancer mortality for the total population in 2020, and 2021 compared to 2015-2019 (per 100,000 population) in 9 countries of the C-MOR consortium**

| **Country** | **Observed average cumulative mortality rate between 2015-2019** | **Observed cumulative mortality rate in 2020** | **Percent Change in 2020 (2020 vs. 2015-2019)** | **Observed cumulative mortality rate in 2021** | **Percent Change in 2020 (2021 vs. 2015-2019)** |
| --- | --- | --- | --- | --- | --- |
| **Australia** | 9753.72 | 9870.95 | +1.2 | 9875.7 | +1.3 |
| **Austria** | 12078.68 | 12417.35 | +2.9 | 11965.95 | -0.8 |
| **Brazil** | 5417.24 | 5564.10 | +2.7 | 5483.35 | +1.2 |
| **Cyprus** | 8528.4 | 9048.15 | +6.1 | 9181.18 | +7.7 |
| **Georgia** | 9454.64 | 11032.30 | +16.7 | 10139.92 | +7.2 |
| **Northern Ireland** | 12450.02 | 12526.02 | +0.6 | 12594.60 | +1.2 |
| **Scotland** | 15554.21 | 15702.19 | +1 | 15757.92 | +1.3 |
| **Slovenia** | 15642.96 | 16020.33 | +2.4 | 15192.15 | -2.9 |
| **Ukraine** | 9274.57 | 9160.22 | -1.2 | 8875.66 | -4.3 |

*The level of significance could not be estimated, considering that the data available was only two time points.*

*Non-shaded area indicates countries of high-income level.*

*Light-shaded area indicates countries of upper-middle income level.*

*Dark-shaded area indicates countries of low-middle income level.*


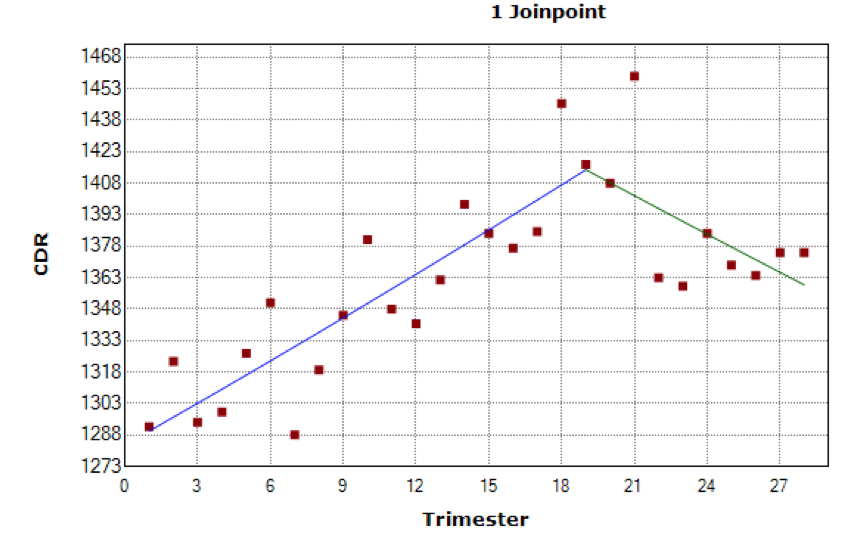

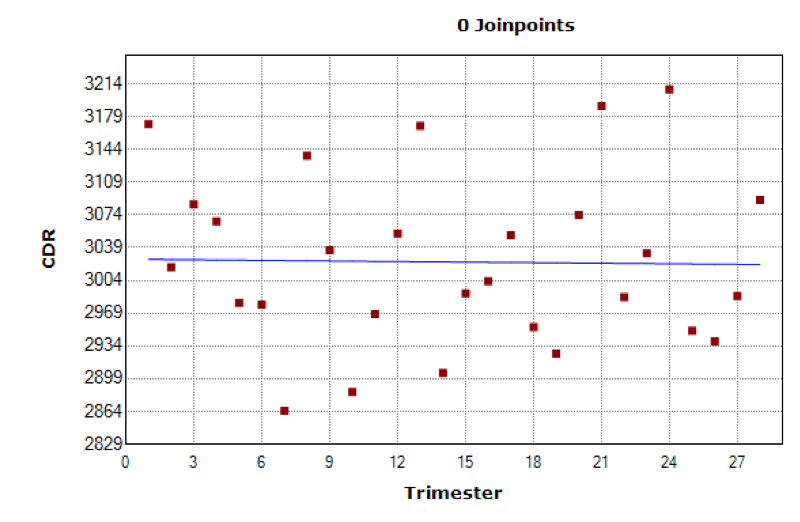

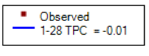
*
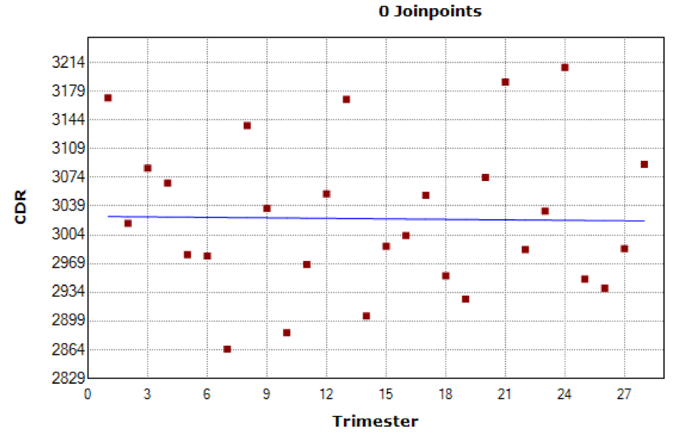
*

**Australia**

**Brazil**

**Austria**


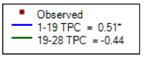
*
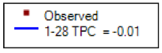
*

**Northern Ireland**

**Georgia**

**Cyprus**

*
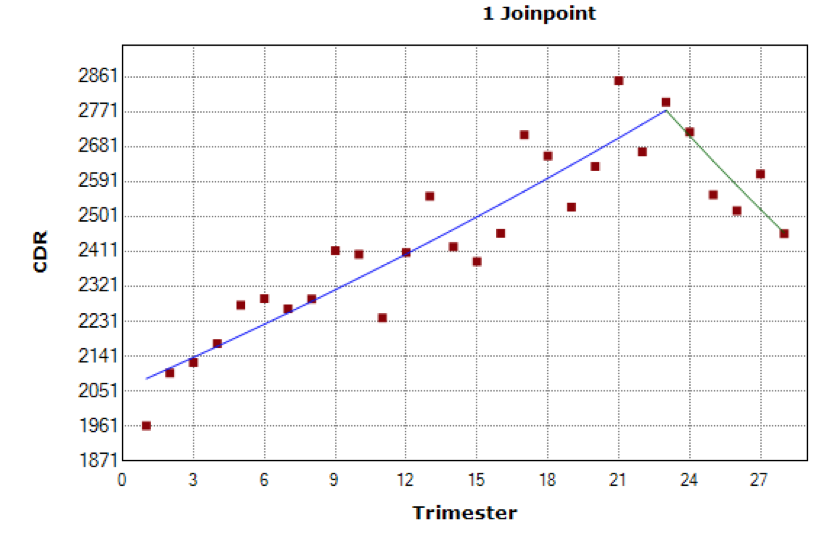
*
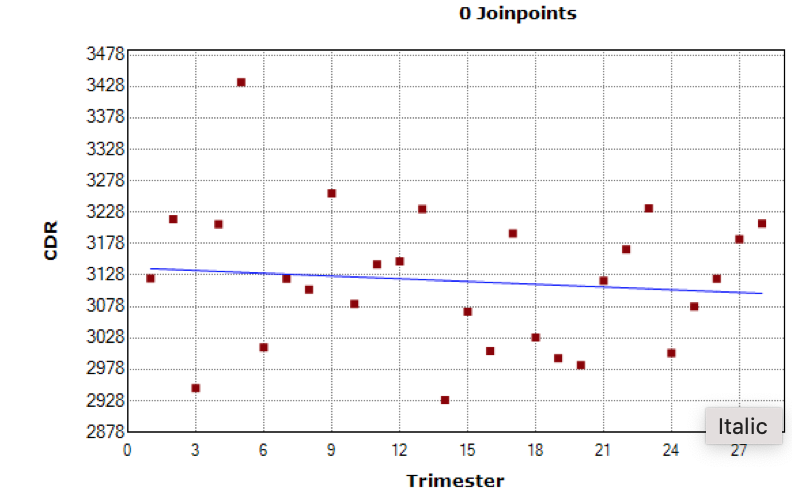

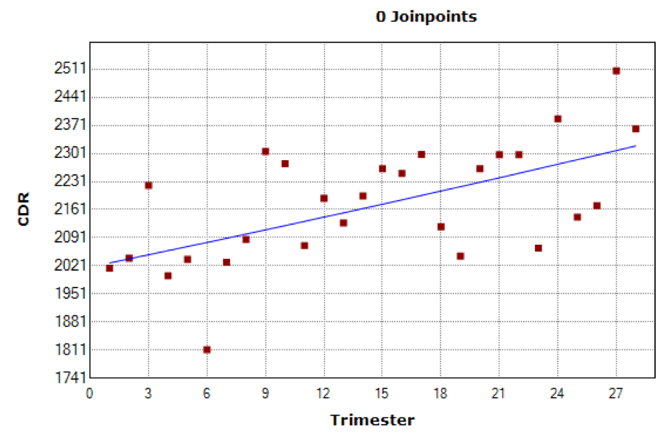


*
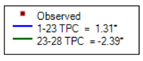

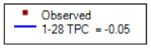

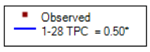
*

**Slovenia**

**Scotland**

*
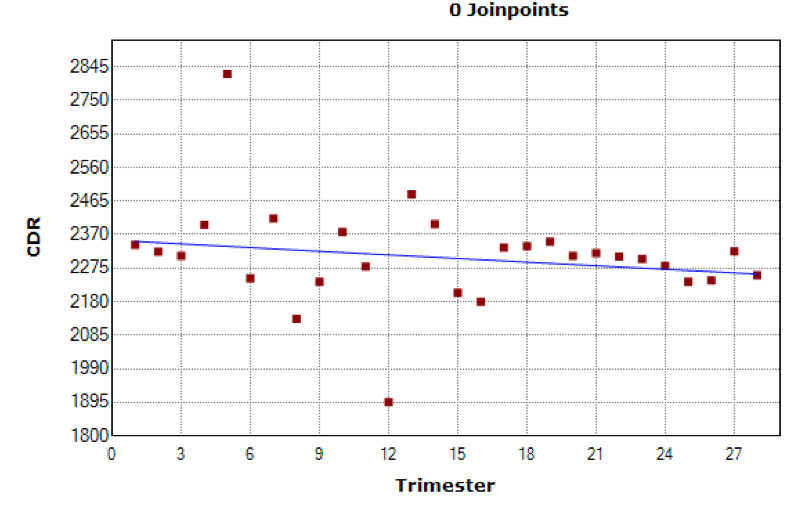

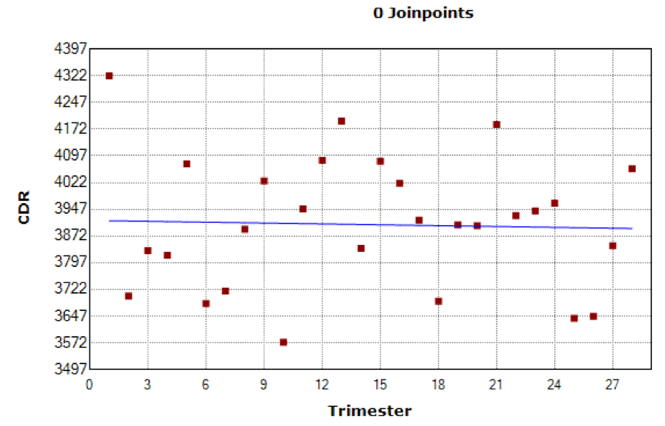

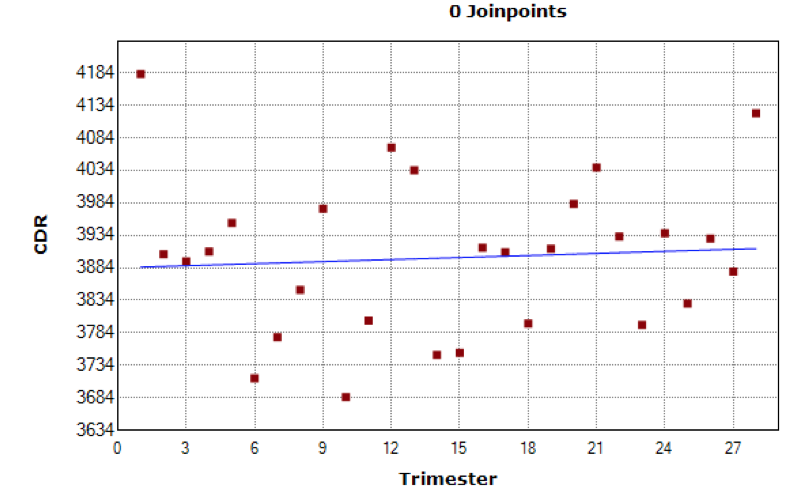
*

**Ukraine**

*
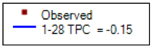
***
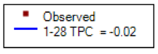
**

*
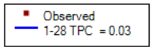
*

**Figure S11. Percent change of cumulative crude cancer mortality rates in 9 countries across trimesters: 1-4 (2015), 5-8 (2016), 9-12 (2017), 13-16 (2018), 17-20 (2019), 21-24 (2020), 25-28 (2021).** **Indicates that the trimester percent change (TPC) is significantly different from zero at the alpha = 0.05 level.*

*The final selected model for each country (number of joinpoints) was determined based on the best fit of the data.*

**Table S9. Summary statistics of pandemic-related variables in 12 countries in 2020 and 2021**

| **Year** | **2020** | | | | | | **2021** | | | | | | | | | |
| --- | --- | --- | --- | --- | --- | --- | --- | --- | --- | --- | --- | --- | --- | --- | --- | --- |
| Country | **Incidence of reported COVID-19 cases per 100,000 population** | | | **Stringency index** | | | **Incidence of reported COVID-19 cases per 100,000 population** | | | **Stringency index** | | | **Vaccination start** | **Fully Vaccinated Individuals** | | |
|  | **Mean** ± **SD** | **Median (IQR)** | **Range** | **Mean** ± **SD** | **Median (IQR)** | **Range** | **Mean** ± **SD** | **Median (IQR)** | **Range** | **Mean** ± **SD** | **Median (IQR)** | **Range** | **2021 Week** | **Mean** ± **SD** | **Median (IQR)** | **Range** |
| Australia | 2302.6 ± 3012.7 | 663.2  (470.5—2671.9) | 380.4—11261.0 | 63.9 ± 14.1 | 68.1  (63.4—71.2) | 19.4—75.5 | 52501.5 ± 179943.4 | 2156.3  (440.2—25849.8) | 416.0—1091750.6 | 61.3 ± 10.6 | 63.3  (52.8—71.8) | 36.6—74.5 | 7 | 29.3 ± 28.6 | 19.2(2.02—58.3) | 0.1—75.6 |
| Austria | 25487.7 ± 34504.4 | 7635.7  (2611.1— 40075.8) | 1536.8—  117700.5 | 54.8 ± 19.7 | 50.5  (38—74.6) | 11.1—82.4 | 30821.4 ± 28368.8 | 24968.9 (11044.3—33977.9) | 2830.9—114957.6 | 61.3 13.3 | 54.6(49.6-76) | 43.2--82.4 | 1 | 35.6 ± 27.2 | 40.7(7--62) | 0.1-70.64 |
| Brazil | 1566604 ± 669483.5 | 1659966 (1121024—2162991) | 34911.1—  2461568.2 | 66.8 ± 16.8 | 71.4  (62.6—77.3) | 5.6—81 | 2091554 ± 1695643 | 1958625 (914194.3—2824175) | 367387.7—11377486.4 | 59.6 ± 8.3 | 60.1 (55.2—66.8) | 44.8—73.6 | 3 | 25.8 ± 23 | 16.6(7—46.6) | 0.01—66.6 |
| Cyprus | 996.2 ± 1429.5 | 248.0  (99.4—1431.6) | 47.7 – 5135.77 | 63.7 ± 19.4 | 63  (50—76.1) | 6.4—94.4 | 4267 ± 8252.6 | 2031.6 (1093.4—4377.3) | 339.2– 53528.4 | 58.7 ± 12.8 | 53.2 (47.8—66.8) | 46.3—84.3 | 1 | 38.4 ± 25.5 | 48.3(8—62.6) | 0.23 –  67.84 |
| England and Wales | 183156.6 ±168630.5 | 131927.5(30233.7—  298984) | 15623 - 592536.3 | 64.8 ± 17.2 | 67.6(65.3—71.7) | 11.1—79.6 | 418372.2 ±760242.4 | 250068.7(71798.6—  326052.6) | 24068.9– 4313129 | 57.5± 17.1 | 51.4(43.6—68.7) | 40—88 | 1 | 41 ± 26.9 | 50.5(11.3—66.5) | 0.7—70.1 |
| Georgia | 37866.6± 57089.2 | 3091.8(1880.9—66330) | 1535.4—172856.2 | 68.3 ± 18.5 | 61.3(57.1—84.3) | 21—100 | 71290.6 ± 60814.3 | 50604.5(30943—92136.8) | 20425 –  336461.8 | 55.5±16.4 | 50.9(45.4—64.8) | 25—84.3 | 13 | 13.6 ± 11.4 | 11.5(2.6—25.1) | 0.16—30.7 |
| Kazakhstan | 107268.8 ± 99492. | 76380.4(20407.2—157975.9) | 1196.1 – 367137.6 | 73 ± 21.2 | 79.6(71.5—  82.4) | 8.3—92.1 | 201712.7 ± 186287.8 | 133984.3 (74102—  242705.3) | 17844.5— 827839 | 67.1 ± 4.1 | 67.3(65.3—  68.7) | 60—75.2 | 6 | ± |  |  |
| Northern Ireland | 174207.7 ± 160007.1 | 118765.7  (29549.5—293507.8) | 15623—  592536.3 | 64.7 ± 17.4 | 67.6(65.2—71.8) | 11.1—79.6 | 345525.5 ± 514081.6 | 250068.7(71798.6—326052.6) | 24068.9— 3073777 | 58.4± 17.3 | 51.4(43.7—70.4) | 40—88 | 2 | 39.1 ± 25.8 | 46.6(10—63.5) | 1.1—68.1 |
| Scotland | 174207.7 ± 160007.1 | 118765.7 (29549.5—293507.8) | 15623—592536.3 | 64.6 ± 17.4 | 67.6 (65.2—71.8) | 11.1—  79.6 | 345525.5 ± 514081.6 | 250068.7(71799—326052.6) | 24068.9 – 3073777 | 58.4 ± 17.3 | 51.4(43.6—70.4) | 40—88 | 1 | 41.5 ± 28.7 | 50(8.9—79) | 0.05—73.1 |
| Slovenia | 6072.4 ± 9083.3 | 703.3(419.5—11013.1) | 147—25488.6 | 59.2 ± 20.4 | 50 (45.5—78) | 9.5—89.8 | 7739.1 ± 7306.1 | 6056.2(2498.1—10876) | 513.7—39974.4 | 54.4±14 | 52(42.8—66.5) | 35.8—82.5 | 2 | 29.4±20.5 | 34.9(6.2—48.8) | 0.01—  55.9 |
| Ukraine | 411455.8 ± 441851 | 172881.1(70358.12—708365.6) | 1357.46 –  1427350 | 64 ± 18.1 | 61.6(58—80) | 9.5—88.9 | 419883.9 ± 286596.1 | 380100.5 (146720.6 —595089.6) | 64641.05—1041554 | 55.1 ± 7.5 | 54.6(51—58.4) | 39.5—71.6 | 8 | 13 ± 11.2 | 11.4(3.1—19.7) | 0.05—34.7 |
| USA | 1236525 ± 928172 | 908385.8(707870.5—1170614) | 66460.72—3544168 | 66.2 ± 14.1 | 69(66.2—72.7) | 8.3—75.5 | 3221929 ± 5950848 | 1535610(1047653—2653582) | 494055.8 –33799888 | 56.4 ± 7.4 | 54.2(52.1—59.5) | 46.7—  71.8 | 1 | 40.8 ± 21 | 50(24.1—58) | 0.35 - 63.3 |

*Non-shaded area indicates countries of high-income level*

*Light-shaded area indicates countries of upper-middle income level*

*Dark-shaded area indicates countries of low-middle income level*
